# Synthesis of New 6-{[ω-(Dialkylamino(heterocyclyl)alkyl]thio}-3-R-2*H*-[1,2,4]triazino[2,3-*c*]quinazoline-2-ones and Evaluation of their Anticancer and Antimicrobial Activities

**DOI:** 10.3797/scipharm.1111-15

**Published:** 2011-12-23

**Authors:** Galina G. Berest, Olexiy Y. Voskoboynik, Sergiy I. Kovalenko, Inna S. Nosulenko, Lyudmyla M. Antypenko, Olexii M. Antypenko, Volodymyr M. Shvets, Andriy M. Katsev

**Affiliations:** 1 Department of Pharmacy, Zaporozhye State Medical University, Mayakovsky ave., 26, 69035, Zaporozhye, Ukraine; 2 Department of Pharmacy, Crimean State Medical University, av. Lenina, 5/7, 95006, Simferopol, Ukraine

**Keywords:** 2*H*-[1,2.4]Triazino[2,3-*c*]quinazolin-2-one, Bioluminescence inhibition, Chemotherapeutic, Antibacterial, Anticancer, Cytostatic, COMPARE, SAR

## Abstract

Several novel 6-thio-3-R-2-oxo-2*H*-[1,2,4]triazino[2,3-*c*]quinazoline-based compounds containing an ω-(dialkylamino(heterocyclyl)]alkyl fragment were synthesized to examine their anticancer activity. Some of the 6-{[ω-(hetero-cyclyl)alkyl]thio}-3-R-2*H*-[1,2,4]triazino[2,3-*c*]quinazoline-2-ones (**3.1–3.10**) were obtained by the nucleophilic substitution of 6-[ω-halogenalkyl]thio-3-R-2*H*-[1,2,4]triazino[2,3-*c*]quinazoline-2-ones (**2.1–2.8**) with azaheterocycles. Alternatively, compounds **3.1–3.22** were synthesized by alkylation of 3-R-6-thio-2*H*-[1,2,4]triazino[2,3-*c*]quinazoline-2-ones potassium salts (**1.1–1.4**) with (2-chloroethyl)-*N*,*N*-dialkylamine hydrochlorides or 1-(2-chloroethyl)heterocycle hydrochlorides. The structures of compounds were elucidated by ^1^H, ^13^C NMR, LC–MS and EI-MS analysis. Then anticancer and antibacterial, bioluminescence inhibition of *Photobacterium leiognathi* Sh1 activities of the substances were tested *in vitro*. It was found that compound **3.18** possessed a wide range of anticancer activity against 27 cell lines of cancer: non-small cell lung, colon, CNS, ovarian, renal, prostate, breast, melanoma and leukemia (log GI_50_ < −5.65). The “structure-activity” relationship was discussed. COMPARE analysis for synthesized anticancer active compounds was performed.

## Introduction

The quinazoline skeleton is a heterocyclic system that can be found in many prospective anticancer drugs [1–17]. Some derivatives, notably 4-R-phenylaminoquinazolines (drugs «Iressa» (**I**), «Erlotinib» (**II**), «Vandetanib» (**III**) and others) are ATP-competitive irreversible inhibitors of the tyrosine-kinase epidermal growth factor receptor and others protein-kinase enzymes, which are widely used in oncological practice (Scheme 1) [3, 5]. It’s important that anticancer activity of 4-R-phenylaminoquinazolines is determined by both the base heterocycle and aniline fragment in 4^th^ position of molecule and requires the presence of halogens, hydroxy and cyano group. To improve the pharmacokinetic properties, notably bioavailability and lipophilicity, it is advisable to introduce appropriate functional groups in 6^th^ and 7^th^ position of molecule. Such anintroduction of acryl- or butyn-2-amide fragments leads to the increase of metabolism and elimination and decrease of accumulation, while methoxy- and heterocyclylalkyloxy-groups are necessary for the improvement of hydrophobic interaction with the appropriate enzymes.

The variants of annelation or introduction of different heterocyclic pharmacophores to quinazoline skeleton were described recently. [18–21]. Thus, [1,2,4]triazino[2,3-*c*]quinazoline (**IV**) is a product of annelation of quinazoline to triazine system, and from the view of medicinal chemistry optimization is undoubtedly an optimal target for anticancer drugs design. So, it is possible to modify the above mentioned molecule at the 3rd position of triazine system, the 6th position of pyrimidine, and the 8th–11th positions of benzene ring. In previous work we’ve made an attempt to modify structure (**IV**) at the 3rd (**V**) and 6th (**VI, VII**) positions [18–22]. Hence, 3-R-6-thioxo-6,7-dihydro-2*H*-[1,2,4]triazino[2,3-*c*]quinazolin-2-one (**VI**) is a promising “lead-compound” to combine with dialkylamino(heterocyclyl)alkyl substituent for development of the potent chemotherapeutic agents.

## Results and discussion

### Chemistry

The known synthetic methods of ω-dialkylamino(heteryl)alkylthioheterocyclic fragment formation are based on two main approaches: the first approach is the interaction of ω-halogenalkylthioheteryles with the excess of secondary amines in anhydrous medium, and the second approach is the direct alkylation of heterylthiones by ω-dialkylamino-alkylhalogenes or 1-(ω-halogenoalkyl)heterocyclyles in organic solvents with the presence of organic or inorganic base.

Alkylation of substances **1.1–1.4** by symmetrical and asymmetrical dihalogenalkanes was investigated to fulfill the first approach. According to the LC-MS data, refluxing the substances **1.1–1.2** with 1,2-dibromoethane for 10–20 min in water-alcohol solution led to the mixture of substances **2.1, 2.2** (up to 90%) with appropriate bisderivatives (4–8%) and starting substances (up to 2–6%). The treatment of compounds **1.3–1.4** with asymmetrical dihalogenalkanes under mentioned conditions yielded 90% of substances **2.1**–**2.8**. When synthesis proceeded longer (up to 60 min) in anhydrous environment (propanol-2, dioxane), that led to less of a yield (up to 60%) with an increase in the amount of bisderivatives (up to 20–40%). The latter effect was caused by the low solubility of potassium salts **1.1–1.4** in anhydrous solvents. It’s important that substances **2.1–2.8** were easily purified by recrystallisation from ethanol or propan-2-ol.

Refluxing of the 6-[ω-halogenalkyl]thio-3-R-2*H*-[1,2,4]triazino[2,3-*c*]quinazoline-2-ones (**2.1**–**2.8**) with the proper equimolar amount of heterocycles (pyrrolidine, piperidine, morpholine) in dioxane with potassium iodide, further neutralizing the obtained hydrochlorides by sodium hydrocarbonate and extraction of substances with chloroform effected the lessening of yields (10–20%) of compounds **3.1**–**3.10** (Scheme 2). Products were quite easily and strongly oxidized in such conditions (reaction mixture became dark brown) that had a substantial influence on the purity of compounds **3.1**–**3.10**. Replacement of solvent by ethanol or propan-2-ol and prolongation of reaction didn’t influence the increasing of substances yields. It was found that the product’s yields and purity could be improved by the addition of the excess heterocycles and refluxing in propan-2-ol–water mixture. Additionally, the obtained amines **3.1–3.10** could be easily extracted from reaction mixture with diethyl ether or chloroform after poured into water.

The 3-R-6-thio-6,7dihydro-2*H*-[1,2,4]triazino[2,3-*c*]quinazoline-2-ones (**1.1**–**1.4**) potassium salts were alkylated with (2-chloroethyl)-*N*,*N*-dialkylamines hydrochlorides or 1-(2-chloro-ethyl)heterocycles hydrochlorides in the presence of triethylamine to fulfill the second approach (Scheme 3). The water–propan-2-ol reaction medium provided the higher yields of resulting products **3.1**–**3.3**, **3.11**–**3.22**.

The individuality and structure of synthesized substances were con rmed by their IR, ^1^H, ^13^C NMR, and EI-MS data. In IR spectra the triazinoquinazoline system was characterized by: ν_C=O_ (1759–1658 cm^−1^), ν_C-C_ (1520cm^−1^ and 1449 cm^−1^), ν_CS_ (713–604 cm^−1^), ν_CS_ and ν_CN_ (1593–710 cm^−1^) and δ_CH_ (902–649 cm^−1^). The symmetric and asymmetric stretchings of CH_2_- moiety of compounds **2.1–2.8**, **3.1–3.22** were detected at 2998–2800 cm^−1^ and deformation vibrations – at 1496–1470 cm^−1^. Additionally long-chain alkanes (**2.3–2.8**, **3.4–3.10**) were characterized by vibrations at 769–760 cm^−1^. Stretching vibration of ν_C-Br_ was characteristic for substances **2.1**, **2.2**, and for substances **2.3–2.8** – ν_C-Cl_ at 750–700 cm^−1^. C-N group of alkylamines chain was confirmed by stretching vibrations at 1395–1001 cm^−1^ and vibrations of N-H group appeared at 3070–3055 cm^−1^ and 1662–1608 cm^−1^ appropriate.

LC-MS spectra of substances **2**, **3** were characterized by positive ions [M+1] and [M+3]. The latter ones characterized the presence of a sulfur isotope. Furthermore, LC-MS spectra of substances **2.3**–**2.8** had an additional signal caused by ion [M+2], which confirmed the presence of a chlorine atom in the molecule.

In ^1^H NMR spectra of **2.1–2.8**, **3.1–3.22** triazinoquinazoline fragment was characterized by two one-proton triplets of H-10 at 7.69–7.48 ppm and H-9 at 7.99–7.88 ppm and two one-proton doublets of H-8 at 7.86–7.68 ppm and H-11 at 8.56–8.37 ppm. In some cases proton H-10 triplet and H-8 doublets of triazinoquinazoline backbone overlapped (**3.14**, **3.15**, **3.17**–**3.22**) or mentioned protons overlaid the two-proton doublets of phenyl substituent (**2.2, 3.2**), forming multiplet. The *p-*substituted phenyl fragment in the 3rd position of substances **2.2**, **2.4**, **2.8**, **3.1**–**3.6**, **3.8**, **3.10**, **3.12**, **3.16**, **3.20** formed A_2_B_2_-system and could be found as two two-proton doublets at 7.39–7.0 ppm and 8.40–8.20 ppm appropriately. Phenyl radical of substances **2.2**, **2.4**, **2.8**, **3.1**–**3.6**, **3.8**, **3.10**, **3.12**, **3.16**, **3.20** formed sub-spectra consisting of two-proton doublet (H-2 and H-6) at 8.35–8.24 ppm and three-proton multiplet (H-3, H-4 and H-5) at 7.66–7.53 ppm. Characteristic protons of –S-CH_2_-group of **2.1–2.6**, **3.1–3.3, 3.6, 3.11–3.22** were registered as triplet at 2.83–2.67 ppm, and for substances **3.4–3.10** – as multiplet at 3.45–2.34 ppm. For compounds **2.1–2.6** protons of -CH_2_-Hal fixed as triplet at 4.25–3.81 ppm and for substances **2.7** and **2.8** – as multiplet at 3.75–3.74 ppm. Protons of dialkylamino and heterylcyclic fragments according to the length or branching had the proper multiplicity in the spectra.

In ^13^C NMR spectra Carbon signals in 6th and 2nd positions for compounds **3.2**, **3.12**, **3.16** and **3.20** were the most shifted ones and appeared at 159–155 ppm and 160 ppm. Carbon of -SCH_2_ group was the additional confirmation of structure and the reaction’s S-regioselectivity resonating at 32.31–28.70 ppm.

EI-MS spectra of substances were characterized by the molecular ion absence due to its low stability. Formation of F_1_ ([R_1_R_2_N-C_2_H_4_]^+•^) with the strong intensity in spectrum was the main direction of molecule’s fragmentation (compound **3.2** – *m/z* 112 (12,2%), **3.12** – *m/z* 72 (68,2%), **3.16** – *m/z* 100 (17,2%), **3.21** – *m/z* 128 (17.6%). Further for F_1_ rejection of one or two atoms of hydrogen and homolytic fragmentation of C–C-bonds with formation of stable fragments were characteristic. For **3.2** the EI-MS spectrum with [C_2_H_3_N-piperidyl]^+^ (*m/z* 111 (100.0)%)), for **3.12** – with [(Me)_2_NCH_2_]^+^ (*m/z* 58 (100.0%)), for **3.16** – with [(Et)_2_NC_2_H_3_]^+^ (*m/z* 99 (100.0%)), for **3.21** – with [(*i*-Pr)_2_NC_2_H_3_]^+^ (*m/z* 127 (88.8%)), with [C_3_H_7_NCH]^+^ (*m/z* 70 (96.7%)) and with [C_3_H_7_]^+^ (*m/z* 43 (100.0%)) were demonstrated. Also, additional confirmation of structures was the formation of intensive F_2_ [C_6_H_5_CN]^+^ with *m/z* 103 (27.3–10.5%) in the result of the C(2) – C(3) and N(3) – N(4) bonds cleavage of triazinoquinazoline system [18–20].

### Pharmacology and structure-activity relationship

#### Bioluminescence inhibition test

The results of bioluminescence research showed that the majority of compounds appeared to be toxic for bacteria *Photobacterium leiognathi* Sh1 (Table 1). Thus, in chronic action test the highest inhibition activity among 6-[ω-halogenalkyl]thio-3-R-2*H*-[1,2,4]triazino[2,3-*c*]quinazoline-2-ones (**2.1**–**2.8)** showed compounds **2.2**, **2.4**–**2.6**, **2.8**, that had aryl substituent at 3rd position. The elongation of the alkyl fragment from ethyl (**2.1**, **2.2)** to isopropyl (**2.3**–**2.6**) and butyl (**2.7, 2.8**) led to decreased cytotoxicity in chronic action test. Such SAR also was characteristic for compounds **3.1**–**3.22**. 6-[(2-Dialkylaminoethyl)thio]-3-R-2*H*-[1,2,4]triazino[2,3*-c*]quinazoline-2-ones (**3.1**–**3.3**, **3.11**–**3.2**) had the highest biocide activity in the acute and chronic action tests. Noticeably, substances **3.1**–**3.3**, **3.11**–**3.22** possessed biocide activity in concentration of 0.1 and 0.25 mg/mL in the acute and chronic actions tests. It is interesting to mention that some of the synthesized compounds (**3.1**–**3.3**, **3.12**–**3.14**, **3.16**, **3.17**, **3.20**) showed effect of hormesis in chronic action test, inhibiting the intensity of bioluminescence in concentration of 0.25 mg/mL.

Thus, the SAR study revealed that:

1. the most cytotoxic substances against luminescent bacteria *Photobacterium leiognathi* strain Sh1 in acute and chronic test appeared to be compounds **3.1–3.22**;
2. substances **3.1–3.22** demonstrated inhibitive activity with the increase of concentration to 0.1 and 0.25 mg/mL;
3. cytotoxicity of 6-{[ω-(heterocyclyl)alkyl]thio}-3-R-2*H*-[1,2,4]triazino[2,3-*c*]quinazoline-2-ones (**3.1–3.10**) is determined by the length of alkyl moiety and decreases in range Et<Pr<Bu;
4. replacement of heteryl fragment of compounds **3.1–3.3** by dialkylamino group (**3.11–3.22**) does not lead to change of cytotoxic activity in chronic test.

Thus, compounds with the highest cytotoxic activity appeared to be 6-[(2-dialkyl-aminoethyl)thio]-3-R-2*H*-[1,2,4]triazino[2,3*-c*]quinazoline-2-ones (**3.1**–**3.3**, **3.11**–**3.22**), that could be the indicator of potential presence of antifungal, antibacterial or anticancer activity of the mentioned compounds.

#### Antimicrobial and antifungal activities

The results of antimicrobial screening showed that researched substances had significant antimicrobial activity only against cereous bacteria *Mycobacterium luteum* (Table 2). Thus, the highest antibacterial data were established for 6-[(2-dialkylaminoethyl)thio]-3-R-2*H*-[1,2,4]triazino[2,3*-c*]quinazoline-2-ones (**3.1**–**3.3**, **3.11**–**3.22**), that inhibited growth of Gram-positive bacteria *M. luteum* at 7–28 mm. Increasing the concentration of compounds **3.1–3.3**, **3.11–3.22** from 1.0 to 5.0 mg/mL also led to considerable growth of bactericidal activity, while elongation of alkyl substituent (**3.4**–**3.10**) resulted in decreased activity and frequently appeared only in concentration of 5.0 mg/mL. It is significant that researched compounds did not show bactericidal action against *E. coli, St.aureus* and *Candida tenuis.* The only compounds that had antibacterial activity against *St. aureus,* inhibiting its growth at 7–13 mm were **3.12**, **3.13**, **3.17** and **3.18**. It is also interesting that compounds **3.13**, **3.17** and **3.18** caused the late spore formation of *A. niger* at 18–23 mm in concentration 0.5 mg/mL.

The SAR study revealed that:

1. antimicrobial activity of researched compounds is more expressed for 6-{[ω-(dialkyl-amino(heterocyclyl)alkyl]thio}-(**3.1–3.22**) than for corresponding (ω-halogenoalkyl)thio-derivatives of 3-R-2*H*-[1,2,4]triazino[2,3-*c*]quinazoline-2-ones (**2.1–2.8**);
2. the main factor for antimicrobial activity demonstration for substances **2.1–2.8, 3.1–3.22** against *S. aureus* is introduction of the [2-(dialkylamino)ethyl]thio]-substituent in the 6th position, but elongation of radical up to propyl or butyl leads to its significant reduction;
3. antimicrobial activity against *M. luteum* is characteristic for the majority of compounds, and compounds with phenyl, thionyl or *p-*methoxyphenyl substituents at 3rd position have the strongest activity.

So, 6-[(2-dialkylaminoethyl)thio]-3-R-2*H*-[1,2,4]triazino[2,3*-c*]quinazoline-2-ones (**3.1**–**3.3**, **3.11**–**3.22**) had the highest antimicrobial activity against *M. luteum*, testifying the potential presence of antituberculosis activity of the mentioned compounds.

#### Anticancer assay for preliminary in vitro testing

Newly synthesized compounds were selected by the National Cancer Institute (NCI) Developmental Therapeutic Program for the *in vitro* cell line screening to investigate their anticancer activity. Compounds **3.1**, **3.14–3.16, 3.18, 3.21** were submitted and evaluated according to the US NCI protocol [23–28]. The compounds were first evaluated at one dose primary anticancer assay toward or approximately 60 cell lines (concentration 10^−5^ M). The human tumor cell lines were derived from nine different cancer types: leukemia, melanoma, lung, colon, CNS, ovarian, renal, prostate and breast cancers. In the screening protocol, each cell line was inoculated and preincubated for 24–48 h on a microtiter plate. Test agents were then added at a single concentration and the culture was incubated for an additional 48 h. End point determinations were made with a protein binding dye, sulforhodamine B (SRB). Results for each test agent were reported as the percent growth of the treated cells when compared to the untreated control cells. The preliminary screening results are shown in Table 3.

Investigation of the compounds **3.1**, **3.14–3.16, 3.18, 3.21** showed that individual cell lines had different sensitivity towards synthesized compounds in concentration 10^−5^ M (Table 3). Thus, substance **3.1** exhibited cytotoxicity against cell lines of leukemia (CCRF-CEM, HL-60(TB)). Change the phenyl substituent in position 3 (**3.1**) by methyl led to the substantial reduction of activity against cell lines of leukemia. Synthesized compounds that had at the 3rd position *p-*methoxyphenyl and *p-*methylphenyl (**3.21**) group possessed strong cytotoxicity against the majority of the cancer cell lines. It is interesting that the significant antiproliferative activity was characteristic for compound **3.18** against leukemia cell lines (K-562, MOLT-4, RPMI-8226, SR), non-small cell lung cancer (A549/ATCC, HOP-62, HOP-92, NCI-H322M, NCI-H460), colon cancer (COLO 205, HCT-116, HCT-15, HT29, KM12, SW-620), CNS cancer (SF-539, U251), melanoma (LOX IMVI, MALME-3M), ovarian cancer (IGROV1, OVCAR-3, OVCAR-4, OVCAR-8, NCI/ADR-RES), renal cancer (ACHN, RXF 393, SN12C, UO-31), prostate cancer (PC-3, DU-145) and breast cancer (MCF7, MDA-MB-231/ATCC). Furthermore, compounds **3.1**, **3.14**, **3.18** showed antitumor activity, notably **3.1** against leukemia cell lines (CCRF-CEM, HL-60(TB)), **3.14** – against leukemia SR, **3.18** – against leukemia SR and non-small cell lung cancer (A549/ATCC, NCI-H460).

The dose-dependent action in 5 concentrations according to standard procedure of NCI (100μM–0.01μM) was researched for **3.14**, **3.16**, **3.18**. The 3 dose-dependent parameters were calculated: 1) GI_50_ – molar concentration of the compound that inhibits 50% net cell growth; 2) TGI – molar concentration of the compound leading to total inhibition of cell growth; 3) LC_50_ – molar concentration of the compound leading to 50% net cell death. If logarithmic data of researched parameters (log GI_50_, log TGI ta log LC_50_) was less than −4.00, substances were marked as active. For each of the parameters the average experimental data were calculated (mean graph midpoints, MG_MID) (Table 4).

The parameters of compounds activity against the most sensitive cell lines are shown in the table 5 (log GI_50_ ≤−5.65). It is necessary to mention the selective sensitivity to cell lines of CNS cancer (SF-539, SNB-75), renal cancer (ACHN), melanoma (LOX IMVI) and renal cancer (ACHN) of compounds **3.14**, **3.16** and **3.18**. Thus, **3.14** revealed high level of inhibition (log GI_50_ = −6.07) against cell line SNB-75 of CNS cancer (MG_MID log GI_50_ = −5.48 for 55 cell lines), **3.16** (log GI_50_ = −6.29) – against cell line A498 of renal cancer (MG_MID log GI_50_ = −5.52 for 59 cell lines), **3.18** (log GI_50_ = −6.20) – against cell line HOP-92 of NSC lung cancer (MG_MID log GI_50_ = −5.57 for 59 cell lines).

**Fig. 1:**
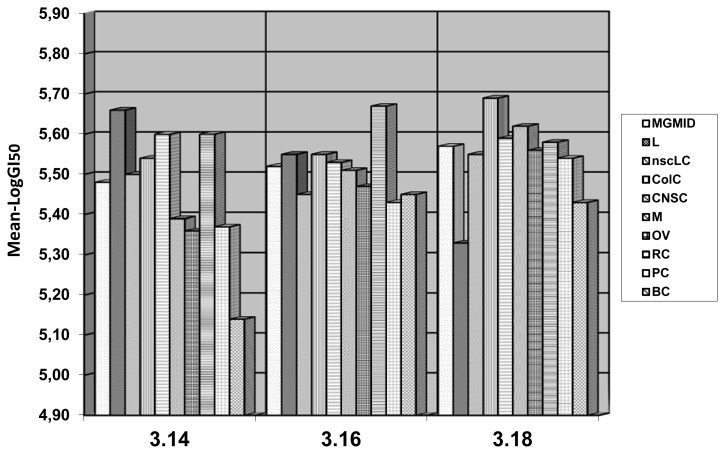
Anticancer selectivity pattern of the most active compounds **3.14**, **3.16** and **3.18**.

It is important to note that compound **3.14** had the highest anticancer activity against cell lines of leukemia (LogGI_50_ = −5.66), CNS cancer (LogGI_50_ = −5.60), renal cancer (LogGI_50_ = −5.60); **3.16** – against cell lines of renal cancer (LogGI_50_ = −5.67); **3.18** – against cell lines of colon cancer (LogGI_50_ = −5.69), CNS cancer (LogGI_50_ = −5,59), melanoma (LogGI_50_ = −5.62), renal cancer (LogGI_50_ = −5.58) (Figure 1).

SAR study revealed that 3-R-6-thio-2*H*-[1,2,4]triazino[2,3-*c*]quinazoline-2-ones derivatives are the class of insufficiently studied annelated quinazolines with triazines substances with promising anticancer activity. It is important that antitumor activity of compounds **3.1**, **3.14–3.16, 3.18, 3.21** is determined by:

1. the heterocycle skeleton;
2. substituent at the 3rd position with increasing activity in the range of Me<Ph<4-MePh<4-MeOPh;
3. substituents at the 6th position, namely dialkylamino(heterocyclil)alkyl fragment connected by Sulphur with heterocyclic system, gaining the activity in range of Me<*i*-Pr<Pyr<Et. Thus, it is interesting to modify the «lead-compounds» among the [1,2,4]triazino[2,3-*c*]quinazoline derivatives for further pharmacological investigations.

### COMPARE analysis and molecular mechanism assumptions

We have also performed COMPARE analyses for all the active compounds to investigate the similarity of their cytotoxicity (mean graph fingerprints) with those of known anticancer standard agents, NCI active synthetic compounds and natural extracts, which are present in public available databases [29–32]. Such analysis is based on comparing the patterns of differential growth inhibition for cultured cell lines and can potentially gain insight into the mechanism of the cytotoxic action. It determines Pearson correlation coefficient (PCC) for the degree of similarity of mean graph fingerprints obtained from *in vitro* anticancer screen with patterns of activity of standard agents. We performed COMPARE computations for synthesized compounds against the NCI ‘Standard Agents’ database at the GI_50_ level (correlations PCC >0.4) (Table 6).

COMPARE analysis hypothesis precludes that the compounds **3.14**, **3.16** and **3.18** might have the same mechanism of action as the agent with known action mechanism, if the data pattern of a compound correlates well with the data pattern of compounds belonging to the standard agent database. The majority of significant correlations for 3-R-6-thio-2*H*-[1,2,4]triazino[2,3-*c*]quinazoline-2-ones derivatives were found with inhibitor of topoisomerases I and II, as well as inhibition or promotion of microtubules polymerization and CTP-synthase inhibitor. These molecular targets should be considered as the first priority and be explored for the leukemia, colon, CNS, renal cancers cell lines.

## Experimental

### Chemistry

#### General methods

Melting points were determined in open capillary tubes and were uncorrected. The elemental analyses (C, H, N, S) were performed using the ELEMENTAR vario EL Cube analyzer (USA). Analyses were indicated by the symbols of the elements or functions within ±0.3% of the theoretical values. IR spectra (4000–600 cm^−1^) were recorded on a Bruker ALPHA FT-IR spectrometer (Bruker Bioscience, Germany) using a module for measuring attenuated total reflection (ATR). ^1^H NMR spectra (500 MHz) and ^13^C NMR spectra (100 MHz): were recorded on a Varian-Mercury 400 (Varian Inc., Palo Alto, CA, USA) spectrometers with TMS as internal standard in DMSO-*d_6_* solution. LC-MS were recorded using chromatography/mass spectrometric system which consists of high performance liquid chromatograph «Agilent 1100 Series» (Agilent, Palo Alto, CA, USA) equipped with diode-matrix and mass-selective detector «Agilent LC/MSD SL» (atmospheric pressure chemical ionization – APCI). Electron impact mass spectra (EI-MS) were recorded on a Varian 1200 L instrument at 70 eV (Varian, USA). The purity of all obtained compounds was checked by ^1^H-NMR and LC-MS.

Substances **1.1–1.4** were synthesized according to the reported procedures [18, 21]. Other starting materials and solvents were obtained from commercially available sources and used without additional purification.

##### General procedure for synthesis of 6-[(ω-halogenoalkyl)thio)-3-R-2H-[1,2,4]triazino-[2,3-c]quinazolin-2-ones (**2.1–2.8**)

To a 0.01 M solution of 3-R-6-thio-6,7dihydro-2*H*-[1,2,4]triazino[2,3-*c*]quinazoline-2-one potassium salt **(1.1–1.4**) in 20 ml of propan-2-ol–water mixture (5:1) was added a solution of 0.01 M of 1,2-dibromoethane, 1-bromo-3-chloropropane or 1-bromo-4-chlorobutane in 10 ml of propan-2-ol. Reaction mixture was heated for 10–20 min, cooled, precipitate was filtered and dried. Formed precipitates were recrystallized from ethanole and propan-2-ol.

###### 6-[(2-Bromoethyl)thio]-3-methyl-2H-[1,2,4]triazino[2,3-c]quinazolin-2-one (**2.1**)

Yield: 55.7%, M.p. 196–200°C; IR (cm^−1^): 3106, 2917, 2850, 2533, 1727, 1659, 1618, 1605, 1582, 1517, 1482, 1376, 1341, 1297, 1258, 1196, 1156, 1107, 1058, 982, 960, 881, 811, 773, 750, 726, 682, 666, 615; ^1^H-NMR (500 MHz, DMSO-d6, TMS): δ=2.36 (s, 3H, CH_3_), 3.38 (t, 2H, -S-C*H*_2_-), 4.20 (t, 2H, -S-CH_2_-C*H*_2_-), 7.48 (t, 1H, *J^3^* = 7.7, H-10), 7.68 (d, 1H, *J* = 7.9, H-8), 7.88 (t, 1H, *J^3^* = 7.7, *J^4^* = 1.4, H-9), 8.43 (d, 1H, *J* = 7.9, H-11); Anal. calcd. for C_13_H_11_BrN_4_OS: C, 44.46; H, 3.16; Br, 22.75; N, 15.95; S, 8.13; Found: C, 44.44; H, 3.13; Br, 22.74; N, 15.96; S, 8.14.

###### 6-[(2-Bromoethyl)thio]-3-phenyl-2H-[1,2,4]triazino[2,3-c]quinazolin-2-one (**2.2**)

Yield: 72.9%, M.p. 192–195°C; IR (cm^−1^): 3096, 2948, 2922, 2861, 1644, 1623, 1606, 1572, 1538, 1494, 1475, 1439, 1373, 1347, 1298, 1281, 1256, 1234, 1178, 1134, 1114, 1080, 1047, 1030, 1002, 986, 942, 883, 872, 858, 814, 768, 756, 695, 656, 607; ^1^H-NMR (500 MHz, DMSO-d6, TMS): δ=3.52 (t, 2H, -S-C*H*_2_-), 4.25 (t, 2H, -S-CH_2_-C*H*_2_-), 7.53–7.48 (m, 4H, H-3′, 5′ 3-Ph, H-8, 10), 7.76–7.67 (m, 2H, H-4′ 3-Ph, H-9), 8.31 (d, 2H, *J*=7.8, H-2′,6′3-Ph), 8.37 (d, 1H, *J*=7.9, H-11); Anal. calcd. for C_18_H_13_BrN_4_OS: C, 52.31; H, 3.17; Br, 19.33; N, 13.56; S, 7.76; Found: C, 52.32; H, 3.19; Br, 19.34; N, 13.58; S, 7.78.

###### 6-[(3-Chloropropyl)thio]-3-methyl-2H-[1,2,4]triazino[2,3-c]quinazolin-2-one (**2.3**)

Yield: 87.5%, M.p. 199–201°C; IR (cm^−1^): 1660, 1625, 1603, 1580, 1556, 1499, 1466, 1429, 1362, 1342, 1311, 1285, 1261, 1222, 1210, 1132, 1104, 1060, 1043, 996, 954, 884, 863, 772, 700, 686, 655, 631, 607; ^1^H-NMR (500 MHz, DMSO-d6, TMS): δ=2.25 (qui, 2H, *J*=6.4, -S-CH_2_-C*H*_2_-), 2.38 (s, 3H, CH_3_), 3.38 (t, 2H, *J*=6.4, -S-C*H*_2_-), 3.82 (t, 2H, *J*=6.4, -S-CH_2_-CH_2_-C*H*_2_-), 7.66 (t, 1H, *J*=7.7, H-10), 7.76 (d, 1H, *J*=7.9, H-8), 7.96 (t, 1H, *J*=7.7, H-9), 8.46 (d, 1H, *J*=7.9, H-11); LC-MS, *m/z* = 321 [M+1], 324 [M+4]; Anal. calcd. for C_19_H_15_ClN_4_OS: C, 52.42; H, 4.08; Cl, 11.05; N, 17.46; S, 9.99; Found: C, 52.42; H, 4.09; Cl, 11.04; N, 17.43; S, 9.98.

###### 6-[(3-Chloropropyl)thio]-3-phenyl-2H-[1,2,4]triazino[2,3-c]quinazolin-2-one (**2.4**)

Yield: 84.9%, M.p. 168–170°C; IR (cm^−1^): 3055, 2924, 1659, 1586, 1566, 1550, 1502, 1486, 1466, 1442, 1398, 1372, 1339, 1310, 1284, 1264, 1234, 1215, 1183, 1157, 1135, 1100, 1078, 1030, 1002, 988, 959, 939, 873, 852, 811, 764, 747, 706, 685, 667, 651, 631; ^1^H-NMR (500 MHz, DMSO-d6, TMS): δ=2.27 (qui, 2H, *J*=6.4, -S-CH_2_-C*H*_2_-), 3.40 (t, 2H, *J*=6.4, -S-C*H*_2_-), 3.84 (t, 2H, *J*=6.4, -S-CH_2_-CH_2_-C*H*_2_-), 7.63–7.56 (m, 3H, H-3′,4′, 5′ 3-Ph), 7.68 (t, 1H, *J*=7.7, H-10), 7.79 (d, 1H, *J*=7.9, H-8), 7.97 (t, 1H, *J*=7.7, H-9), 8.28 (d, 2H, *J*=7.3, H-2′,6′ 3-Ph), 8.48 (d, 1H, *J*=7.9, H-11); LC-MS, *m/z* = 383 [M+1], 385 [M+3], 386 [M+4]; Anal. calcd. for C_19_H_15_ClN_4_OS: C, 59.60; H, 3.95; Cl, 9.26; N, 14.63; S, 8.37; Found: C, 59.60; H, 3.95; Cl, 9.24; N, 14.63; S, 8.37.

###### 6-[(3-Chloropropyl)thio]-3-(4-methylphenyl)-2H-[1,2,4]triazino[2,3-c]quinazolin-2-one (**2.5**)

Yield: 84.7%, M.p. 179–182°C; IR (cm^−1^): 3245, 2923, 1761, 1661, 1626, 1610, 1587, 1562, 1549, 1497, 1469, 1403, 1367, 1339, 1307, 1266, 1241, 1185, 1156, 1139, 1108, 1074, 1021, 989, 964, 940, 880, 856, 833, 771, 713, 686, 641, 627; ^1^H-NMR (500 MHz, DMSO-d6, TMS): δ=2.28 (qui, 2H, *J*=6.4, -S-CH_2_-C*H*_2_-), 2.42 (s, 3H, C*H*_3_), 3.42 (t, 2H, *J*=6.4, -S-C*H*_2_-), 3.84 (t, 2H, *J*=6.4, -S-CH_2_-CH_2_-C*H*_2_-), 7.39 (d, 2H, *J*=7.9, H-3′,5′ Ph)), 7.68 (t, 1H, *J*=7.7, H-10), 7.79 (d, 1H, *J*=7.9, H-8), 7.97 (t, 1H, *J*=7.7, H-9), 8.23 (d, 2H, *J*=7.9, H-2′,6′ Ph), 8.49 (d, 1H, *J*=7.9, H-11); LC-MS, *m/z* = 397 [M+1], 399 [M+3], 400 [M+4]; Anal. calcd. for C_20_H_17_ClN_4_OS: C, 60.52; H, 4.32; Cl, 8.93; N, 14.12; S, 8.08; Found: C, 60.51; H, 4.30; Cl, 8.94; N, 14.12; S, 8.06.

###### 6-[(3-Chloropropyl)thio]-3-(4-methoxyphenyl)-2H-[1,2,4]triazino[2,3-c]quinazolin-2-one (**2.6**)

Yield: 84.8%, M.p. 177–183°C; IR (cm^−1^): 3234, 2973, 2924, 2894, 2838, 1761, 1657, 1630, 1603, 1585, 1562, 1541, 1489, 1470, 1453, 1409, 1385, 1341, 1321, 1303, 1255, 1240, 1173, 1140, 1088, 1046, 988, 941, 908, 879, 853, 837, 809, 758, 715, 705, 682, 636, 623; ^1^H-NMR (500 MHz, DMSO-d6, TMS): δ=2.31 (qui, 2H, *J*=6.4, -S-CH_2_-C*H*_2_-), 3.44 (t, 2H, *J*=6.4, -S-C*H*_2_-), 3.81 (t, 2H, *J*=6.4, -S-(CH_2_)_2_-C*H*_2_-), 3.90 (s, 3H, C*H*_3_O), 7.06 (d, 2H, *J*=8.9, H-3′,5′ Ph), 7.66 (t, 1H, *J*=7.7, H-10), 7.78 (d, 1H, *J*=7.9, H-8), 7.94 (t, 1H, *J*=7.7, H-9), 8.40 (d, 2H, *J*=8.9, H-2′,6′ Ph), 8.53 (d, 1H, *J*=7.9, H-11); LC-MS, *m/z* = 413 [M+1], 415 [M+3], 417 [M+4]; Anal. calcd. for C_20_H_17_ClN_4_O_2_S: C, 58.18; H, 4.15; Cl, 8.59; N, 13.57; S, 7.77; Found: C, 58.18; H, 4.15; Cl, 8.58; N, 13.57; S, 7.77.

###### 6-[(3-Chlorobutyl)thio]-3-methyl-2H-[1,2,4]triazino[2,3-c]quinazolin-2-one (**2.7**)

Yield: 87.5%, M.p. 199–201°C; IR (cm^−1^): 2962, 2910, 1660, 1581, 1557, 1501, 1464, 1425, 1360, 1337, 1309, 1280, 1254, 1219, 1203, 1130, 1101, 1034, 1001, 950, 882, 858, 787, 767, 700, 688, 629, 605; ^1^H-NMR (500 MHz, DMSO-d6, TMS): δ=1.96 (m, 4H, -S-CH_2_- (C*H*_2_)_2_-), 2.39 (s, 3H, CH_3_), 3.33 (m, 2H, -S-C*H*_2_-), 3.74 (m, 2H, -S-(CH_2_)_3_-C*H*_2_-), 7.66 (t, 1H, *J*=7.7, H-10), 7.77 (d, 1H, *J*=7.9, H-8), 7.96 (t, 1H, *J*=7.7, H-9), 8.49 (d, 1H, *J*=7.9, H-11); LC-MS, *m/z* = 335 [M+1], 337 [M+3], 338 [M+4]; Anal. calcd. for C_15_H_15_ClN_4_OS: C, 63.81; H, 4.52; Cl, 10.59; N, 16.73; S, 9.58; Found: C, 63.81; H, 4.52; Cl, 10.58; N, 16.73; S, 9.58.

###### 6-[(3-Chlorobutyl)thio]-3-phenyl-2H-[1,2,4]triazino[2,3-c]quinazolin-2-one (**2.8**)

Yield: 87.5%, M.p. 199–201°C; IR (cm^−1^): 3072, 2937, 2857, 1666, 1585, 1553, 1503, 1487, 1470, 1444, 1404, 1370, 1341, 1312, 1285, 1262, 1243, 1217, 1182, 1137, 1105, 1080, 1022, 1002, 989, 940, 879, 850, 812, 782, 767, 754, 689, 653, 614; ^1^H-NMR (500 MHz, DMSO-d6, TMS): δ=2.06 (m, 4H, -S-CH_2_-(C*H*_2_)_2_-), 3.46 (m, 2H, -S-C*H*_2_-), 3.75 (m, 2H, -S-(CH_2_)_3_-C*H*_2_-), 7.63–7.56 (m, 3H, H-3′,4′, 5′ Ph), 7.69 (t, 1H, *J*=7.7, H-10), 7.80 (d, 1H, *J*=7.9, H-8), 7.99 (t, 1H, *J*=7.7, H-9), 8.29 (d, 2H, *J*=7.3, H-2′,6′ Ph), 8.51 (d, 1H, *J*=7.9, H-11); LC-MS, *m/z* = 397 [M+1], 399 [M+3], 400 [M+4]; Anal. calcd. for C_20_H_17_ClN_4_OS: C, 60.52; H, 4.32; Cl, 8.93; N, 14.12; S, 8.08; Found: C, 60.52; H, 4.33; Cl, 8.94; N, 14.13; S, 8.10.

##### General procedure for synthesis of 6-[(ω-dialkylamino(heterocyclyl-)alkyl)thio]-3-R-2H-[1,2,4]triazino[2,3-c]quinazolin-2-ones (**3.1–3.22**)

###### Method A

To a stirred suspension of 0.01 M of 6-[ω-halogenalkyl]thio-3-R-2*H*-[1,2,4]triazino[2,3-*c*]quinazoline-2-one (**2.1–2.4**, **2.7**, **2.8**) in 20 ml of propan-2-ol or dioxane was added 0.03–0.04 M of proper amine (pyrrolidine, piperidine, morpholine) and 0.01 M of potassium iodide. The resulting mixture was refluxed for 10 h. After cooling the resulting mixture was poured in water, substances **3.1–3.10** were extracted by diethyl ether or chloroform. Organic solvent was removed. Formed precipitates were recrystallized from ethanol.

###### Method B

To a stirred suspension of 0.01 M of potassium salt of 3-R-6-thio-2*H*-[1,2,4]triazino[2,3-*c*]quinazoline-2-one (**2.1–2.6**) in 20 ml mixture of propan-2-ol–water (5:1) or propan-2-ol was added 0.17 ml (0.001M) of triethylamine and 0.01 M of (2-chloroethyl)-*N*,*N*-dialkylamine hydrochloride or of 1-(2-chloroethyl)heterocycle hydrochloride. The resulting mixture was refluxed for 30–90 min. Solution was cooled, poured in water, obtained compounds **3.1**–**3.3**, **3.11**–**3.22** were extracted by diethyl ether or chloroform. Organic solvent was removed. Formed precipitates were recrystallized from ethanol and dried.

###### 3-Phenyl-6-[(2-pyrrolidin-1-ylethyl)thio]-2H-[1,2,4]triazino[2,3-c]quinazolin-2-one (**3.1**)

Yield: Method A, 30.6%; Method B, 70.6%; M.p. 172–174°C; IR (cm^−1^): 2955, 2930, 2874, 2798, 1730, 1662, 1587, 1564, 1550, 1504, 1485, 1469, 1455, 1372, 1341, 1309, 1285, 1266, 1244, 1232, 1180, 1138, 1110, 1078, 1031, 1002, 987, 939, 903, 880, 852, 810, 788, 778, 750, 686, 654, 623; ^1^H-NMR (500 MHz, DMSO-d6, TMS): δ=1.70 (qui, 4H, N(CH_2_)_2_(C*H*_2_)_2_), 2.49 (qui, 4H, N(C*H*_2_)_2_), 2.67 (t, 2H, *J*=6.9, -S-CH*_2_*-C*H*_2_-), 3.41 (t, 2H, *J*=6.9, -S-C*H**_2_*-), 7.65–7.57 (m, 3H, H-3′,4′,5′ Ph), 7.64 (t, 1H, *J*=7.3, H-10), 7.73 (d, 1H, *J*=7.9, H-8), 7.96 (t, 1H, *J*=7.3, H-9), 8.24 (d, 2H, *J*=7.3, H-2′,6′ Ph), 8.44 (d, 1H, *J*=7.9, H-11); LC-MS, *m/z* = 404 [M+1], 406 [M+3]; Anal. calcd. for C_22_H_21_N_5_OS: C, 65.49; H, 5.75; N, 17.36; S, 7.95; Found: C, 65.49; H, 5.75; N, 17.36; S, 7.95.

###### 3-Phenyl-6-[(2-piperidine-1-ylethyl)thio]-2H-[1,2,4]triazino[2,3-c]quinazolin-2-one (**3.2**)

Yield: Method A, 34.3%, Method B, 74.3%; M.p. 150–153°C; IR (cm^−1^): 3055, 2955, 2929, 2850, 2800, 2777, 2754, 2737, 2690, 2667, 2354, 1759, 1729, 1651, 1610, 1583, 1547, 1496, 1483, 1465, 1453, 1444, 1377, 1339, 1322, 1307, 1277, 1258, 1239, 1212, 1182, 1165, 1146, 1135, 1124, 1105, 1079, 1042, 1031, 1021, 1000, 986, 957, 940, 909, 892, 876, 849, 813, 788, 779, 769, 750, 706, 688, 652, 613; ^1^H-NMR (500 MHz, DMSO-d6, TMS): δ=1.40 (qui, 2H, -N(CH_2_CH_2_)_2_C*H**_2_*), 1.53 (qui, 4H, -N(CH_2_C*H*_2_)_2_CH_2_), 2.48 (t, 4H, -N(C*H*_2_CH_2_)_2_CH*_2_*), 2.69 (t, 2H, *J*=6.8, -S-CH_2_-C*H*_2_-), 3.37 (t, 2H, *J*=6.8, -S-C*H*_2_-),7.66–7.56 (m, 4H, H-3′, 4′, 5′ Ph, H-10), 7.70 (d, 1H, *J*=7.9, H-8), 7.94 (t, 1H, *J*=7.7, H-9), 8.25 (d, 2H, *J*=7.3, 2′, 6′ Ph), 8.44 (d, 1H, *J*=7.9, H-11); ^13^C-NMR (100 MHz): δ=24.53 (*C*H_2_(CH_2_CH_2_)_2_N-), 26.04 (CH_2_(*C*H_2_CH_2_)_2_N-), 28.70 (-S-*C*H_2_-), 54.27 (-S-CH_2_-*C*H_2_-), 57.29 (CH_2_(CH_2_*C*H_2_)_2_N-), 118.07 (8), 107.35 (11a), 126.04 (10), 127.87 (11), 129.89 (3′, 5′ Ph), 129.81 (2′, 6′ Ph), 131.94 (4′ Ph, 11), 132.15 (1′ Ph), 136.02 (9), 144.24 (3), 149.56 (11b), 151.02 (7a), 155.49 (2), 160.00 (6); EI-MS, *m/z* (I_rel_, %) = 170 (5.6), 112 (12.2), 111 (100.0), 103 (10.5), 99 (12.1), 98 (68.8), 96 (21.3), 83 (9.8), 76 (5.5), 70 (8.5), 69 (7.8), 56 (8.2), 55 (14.7), 44 (5.6), 42 (8.4), 41 (8.0); LC-MS, *m/z* = 418 [M+1], 420 [M+3]; Anal. calcd. for C_23_H_25_N_5_OS: C, 66.16; H, 5.55; N, 16.77; S, 7.68; Found: C, 66.18; H, 5.57; N, 16.79; S, 7.69.

###### 6-[(2-Morpholin-4-ylethyl)thio]-3-phenyl-2H-[1,2,4]triazino[2,3-c]quinazolin-2-one (**3.3**)

Yield: Method A, 36.0%, Method B, 56.0%; M.p. 212–214°C; IR (cm^−1^): 3070, 2956, 2922, 2855, 2800, 2763, 2737, 2700, 2661, 1727, 1662, 1614, 1582, 1551, 1504, 1482, 1468, 1454, 1376, 1337, 1281, 1258, 1194, 1134, 1115, 1070, 1057, 1041, 1002, 953, 918, 894, 867, 852, 811, 771, 750, 702, 682, 665, 629, 613; ^1^H-NMR (500 MHz, DMSO-d6, TMS): δ=1.17 (qui, 4H, -N(C*H*_2_CH_2_)_2_O), 2.75 (t, 2H, *J*=6.8, -S-CH_2_-C*H*_2_-), 3.09 (qui, 4H, -N(CH_2_C*H*_2_)_2_O), 3.61 (t, 2H, *J*=6.8, -S-C*H*_2_-), 7.62–7.54 (m, 4H, H-3′, 4′, 5′ Ph, H-10), 7.78 (d, 1H, *J*=7.9, H-8), 7.97 (t, 1H, *J*=7.7, H-9), 8.33 (d, 2H, *J*=7.3, 2′, 6′ Ph), 8.49 (d, 1H, *J*=7.9, H-11); LC-MS, *m/z* = 420 [M+1], 422 [M+3]; Anal. calcd. for C_22_H_21_N_5_O_2_S: C, 62.99; H, 5.05; N, 16.69; S, 7.64; Found: C, 62.99; H, 5.05; N, 16.69; S, 7.64.

###### 3-Phenyl-6-[(2-pyrrolidin-1-ylpropyl)thio]-2H-[1,2,4]triazino[2,3-c]quinazolin-2-one (**3.4**)

Yield: Method A, 33.9%; M.p. 146–148°C; IR (cm^−1^): 3056, 2998, 2950, 2929, 2865, 2662, 2563, 2479, 2352, 2318, 1730, 1665, 1586, 1563, 1553, 1504, 1487, 1469, 1443, 1371, 1341, 1312, 1283, 1266, 1242, 1187, 1156, 1135, 1104, 1080, 1022, 1002, 988, 941, 850, 812, 779, 751, 689, 668, 654, 623; ^1^H-NMR (500 MHz, DMSO-d6, TMS): δ=2.03 (m, 4H, -N(CH_2_)_2_(C*H*_2_)_2_), 2.33 (m, 2H, -S-CH_2_-C*H*_2_-), 2.99 (m, 4H, -N(C*H*_2_)_2_(CH_2_)_2_), 3.31 (m, 2H, S-(CH_2_)_2_-C*H*_2_-), 3.58 (m, 2H, -S-C*H*_2_-), 7.60–7.53 (m, 3H, H-3′,4′, 5′ Ph), 7.67 (t, 1H, *J*=7.7, H-10), 7.86 (d, 1H, *J*=7.9, H-8), 7.96 (t, 1H, *J*=7.7, H-9), 8.34 (d, 2H, *J*=7.3, H-2′,6′ Ph), 8.56 (d, 1H, *J*=7.9, H-11); LC-MS, *m/z* = 418 [M+1], 420 [M+3]; Anal. calcd. for C_23_H_23_N_5_OS: C, 66.16; H, 5.55; N, 16.77; S, 7.68; Found: C, 66.18; H, 5.58; N, 16.78; S, 7.69.

###### 3-Phenyl-6-[(2-piperidine-1-ylpropyl)thio]-2H-[1,2,4]triazino[2,3-c]quinazolin-2-one (**3.5**)

Yield: Method A, 55.4%; M.p. 151–152°C; IR (cm^−1^): 2955, 2930, 2874, 2798, 1730, 1662, 1587, 1564, 1550, 1504, 1485, 1469, 1455, 1372, 1341, 1309, 1285, 1266, 1244, 1232, 1180, 1138, 1110, 1078, 1031, 1002, 987, 939, 903, 880, 852, 810, 788, 778, 750, 686, 654, 623; ^1^H-NMR (500 MHz, DMSO-d6, TMS): δ=2.33 (m, 2H, -S-CH_2_-C*H*_2_-), 2.41 (m, 6H, -N(CH_2_)_2_(C*H*_2_)_3_), 3.45 (m, 2H,-S-(CH_2_)_2_-C*H*_2_-), 3.57 (m, 4H, -N(C*H*_2_)_2_(CH_2_)_3_), 3.80 (m, 2H, -S-C*H*_2_-), 7.62–7.53 (m, 4H, H-3′,4′, 5′ Ph, H-10), 7.69 (d, 1H, *J*=7.9, H-8), 7.80 (t, 1H, *J*=7.7, H-9), 8.35 (d, 2H, *J*=7.3, H-2′,6′ Ph), 8.51 (d, 1H, *J*=7.9, H-11); LC-MS, *m/z* = 432 [M+1], 434 [M+2]; Anal. calcd. for C_24_H_25_N_5_OS: C, 66.80; H, 5.84; N, 16.23; S, 7.43; Found: C, 66.82; H, 5.88; N, 16.23; S, 7.44.

###### 6-[(2-Morpholin-4-ylpropyl)thio]-3-phenyl-2H-[1,2,4]triazino[2,3-c]quinazolin-2-one (**3.6**)

Yield: Method A, 50.7%; M.p. 158–160°C; IR (cm^−1^): 3055, 2954, 2925, 2853, 1661, 1589, 1566, 1550, 1502, 1486, 1467, 1442, 1399, 1372, 1340, 1310, 1283, 1264, 1234, 1217, 1180, 1159, 1135, 1117, 1080, 1031, 1021, 1003, 989, 960, 939, 872, 855, 811, 764, 749, 706, 685, 667, 651, 632, 613; ^1^H-NMR (500 MHz, DMSO-d6, TMS): δ=2.31 (qui, 2H, *J*=6.4, -S-CH_2_-C*H*_2_-), 2.54 (m, 4H, -N(C*H*_2_)_2_(CH_2_)_2_O), 3.44 (t, 2H, *J*=6.4, -S-(CH_2_)_2_-C*H*_2_-), 3.59 (m, 4H, -N(CH_2_)_2_(C*H*_2_)_2_O), 3.80 (t, 2H, *J*=6.4, -S-C*H*_2_-), 7.59–7.53 (m, 3H, H-3′,4′, 5′ Ph), 7.66 (t, 1H, *J*=7.7, H-10), 7.76 (d, 1H, *J*=7.9, H-8), 7.94 (t, 1H, *J*=7.7, H-9), 8.34 (d, 2H, *J*=7.3, H-2′,6′ Ph), 8.55 (d, 1H, *J*=7.9, H-11); LC-MS, *m/z* = 434 [M+1], 435 [M+2]; Anal. calcd. for C_23_H_23_N_5_O_2_S: C, 63.72; H, 5.35; N, 16.15; S, 7.40; Found: C, 63.75; H, 5.36; N, 16.16; S, 7.41.

###### 3-Methyl-6-[(2-piperidine-1-ylbutyl)thio]-2H-[1,2,4]triazino[2,3-c]quinazolin-2-one (**3.7**)

Yield: Method A, 56.9%; M.p. >300°C; IR (cm^−1^): 3502, 2916, 2851, 2806, 2768, 1662, 1625, 1601, 1581, 1558, 1503, 1466, 1429, 1362, 1339, 1284, 1260, 1222, 1157, 1132, 1105, 1042, 953, 874, 768, 686, 630, 608; ^1^H-NMR (500 MHz, DMSO-d6, TMS): δ=1.43 (m, 2H, -N(CH_2_)_2_(CH_2_)_2_C*H*_2_), 1.54 (m, 2H, -S-(CH_2_)_2_-C*H*_2_-), 1.66 (m, 4H, -N(CH_2_)_2_(C*H*_2_)_2_CH_2_), 1.81 (qui, 2H, -S-CH_2_-C*H*_2_-), 1.97 (m, 4H, -N(C*H*_2_)_2_(CH_2_)_2_CH_2_), 2.34 (m, 2H, -S-(CH_2_)_3_-C*H*_2_-), 2.43 (s, 3H, CH_3_), 3.29 (qui, 2H, -S-C*H*_2_-), 7.64 (t, 1H, *J*=7.7, H-10), 7.73 (d, 1H, *J*=7.9, H-8), 7.91 (t, 1H, *J*=7.7, H-9), 8.52 (d, 1H, *J*=7.9, H-11); Anal. calcd. for C_20_H_25_N_5_OS: C, 62.64; H, 6.57; N, 18.26; S, 8.36; Found: C, 62.65; H, 6.58; N, 18.25; S, 8.38.

###### 3-Phenyl-6-[(2-piperidine-1-ylbutyl)thio]-2H-[1,2,4]triazino[2,3-c]quinazolin-2-one (**3.8**)

Yield: Method A, 60.3%; M.p. 278–280°C; IR (cm^−1^): 3068, 2936, 1665, 1583, 1552, 1502, 1486, 1468, 1442, 1402, 1371, 1340, 1311, 1283, 1261, 1237, 1181, 1156, 1136, 1103, 1079, 1031, 1021, 1001, 987, 939, 878, 849, 811, 783, 766, 752, 721, 687, 652, 613; ^1^H-NMR (500 MHz, DMSO-d6, TMS): δ=1.43 (m, 2H, -N(CH_2_)_2_(CH_2_)_2_C*H*_2_), 1.55 (m, 2H, -S-(CH_2_)_2_-C*H*_2_-), 1.69 (m, 4H, -N(CH_2_)_2_(C*H*_2_)_2_CH_2_), 1.85 (qui, 2H, -S-CH_2_-C*H*_2_-), 2.00 (m, 4H, -N(C*H*_2_)_2_(CH_2_)_2_CH_2_), 2.37 (m, 2H, -S-(CH_2_)_3_-C*H*_2_-), 3.34 (qui, 2H, -S-C*H*_2_-), 7.59–7.52 (m, 3H, H-3′,4′, 5′ Ph), 7.65 (t, 1H, *J*=7.7, H-10), 7.76 (d, 1H, *J*=7.9, H-8), 7.94 (t, 1H, *J*=7.7, H-9), 8.31 (d, 2H, *J*=7.3, H-2′,6′ Ph), 8.55 (d, 1H, *J*=7.9, H-11); Anal. calcd. for C_25_H_27_N_5_OS: C, 67.39; H, 6.11; N, 15.72; S, 7.20; Found: C, 67.36; H, 6.12; N, 15.73; S, 7.24.

###### 3-Methyl-6-[(2-morpholin-4-ylbutyl)thio]-2H-[1,2,4]triazino[2,3-c]quinazolin-2-one (**3.9**)

Yield: Method A, 56.9%; M.p. >300°C; IR (cm^−1^): 2961, 2911, 2856, 1662, 1625, 1599, 1582, 1559, 1502, 1466, 1429, 1405, 1361, 1339, 1311, 1284, 1258, 1222, 1208, 1132, 1042, 1002, 953, 874, 861, 767, 701, 686, 630, 606; ^1^H-NMR (500 MHz, DMSO-d6, TMS): δ=1.98 (m, 4H, -S-CH_2_-(C*H*_2_)_2_-), 2.45–2.36 (m, 5H, CH_3_, -S-(CH_2_)_3_-C*H*_2_-), 2.54 (m, 4H, -N(C*H*_2_)_2_(CH_2_)_2_O), 3.31 (m, 2H, -S-C*H*_2_-), 3.69–3.57 (m, 4H, -N(CH_2_)_2_(C*H*_2_)_2_O), 7.62 (t, 1H, *J*=7.7, H-10), 7.73 (d, 1H, *J*=7.9, H-8), 7.92 (t, 1H, *J*=7.7, H-9), 8.53 (d, 1H, *J*=7.9, H-11); Anal. calcd. for C_19_H_23_N_5_O_2_S: C, 59.20; H, 6.01; N, 18.17; S, 8.32; Found: C, 59.22; H, 6.03; N, 18.19; S, 8.33.

###### 6-[(2-Morpholin-4-ylbutyl)thio]-3-phenyl-2H-[1,2,4]triazino[2,3-c]quinazolin-2-one (**3.10**)

Yield: Method A, 51.4%; M.p. 278–280°C; IR (cm^−1^): 3058, 3005, 2953, 2914, 2852, 1664, 1587, 1559, 1502, 1486, 1467, 1444, 1370, 1339, 1312, 1283, 1264, 1241, 1229, 1184, 1162, 1135, 1120, 1104, 1080, 1047, 1032, 1021, 1002, 987, 940, 887, 874, 852, 812, 786, 752, 706, 688, 666, 653, 613; ^1^H-NMR (500 MHz, DMSO-d6, TMS): δ=2.00 (m, 4H, -S-CH_2_-(C*H*_2_)_2_-), 2.40–2.33 (m, 2H, -S-(CH_2_)_3_-C*H*_2_-), 2.58 (m, 4H, -N(C*H*_2_)_2_(CH_2_)_2_O), 3.35 (m, 2H, -S-C*H*_2_-), 3.69 (m, 4H, -N(CH_2_)_2_(C*H*_2_)_2_O), 7.60–7.52 (m, 3H, H-3′,4′, 5′ Ph), 7.66 (t, 1H, *J*=7.7, H-10), 7.78 (d, 1H, *J*=7.9, H-8), 7.95 (t, 1H, *J*=7.7, H-9), 8.34 (d, 2H, *J*=7.3, H-2′,6′ Ph), 8.55 (d, 1H, *J*=7.9, H-11); Anal. calcd. for C_24_H_25_N_5_O_2_S: C, 64.41; H, 5.63; N, 15.65; S, 7.16; Found: C, 64.41; H, 5.62; N, 15.63; S, 7.18.

###### 6-{[2-(Dimethylamino)ethyl]thio}-3-methyl-2H-[1,2,4]triazino[2,3-c]quinazolin-2-one (**3.11**)

Yield: Method B, 78.9%, M.p. 144–146°C; IR (cm^−1^): 3410, 3354, 3293, 3066, 2977, 2947, 2917, 2855, 2817, 2762, 2714, 2392, 1666, 1623, 1600, 1581, 1557, 1504, 1481, 1465, 1428, 1376, 1363, 1346, 1312, 1287, 1262, 1223, 1206, 1161, 1133, 1056, 1043, 1015, 953, 885, 850, 771, 738, 686, 630, 606; ^1^H-NMR (500 MHz, DMSO-d6, TMS): δ=2.31 (s, 6H, -N(CH_3_)_2_, 2.37 (s, 3H, CH_3_), 2.79 (t, 2H, *J*=6.9, -S-CH*_2_*-C*H*_2_-), 3.39 (t, 2H, *J*=6.9, -S-C*H**_2_*-), 7.65 (t, 1H, *J*=7.1, H-10), 7.74 (d, 1H, *J*=7.9, H-8), 7.95 (t, 1H, *J*=7.1, H-9), 8.45 (d, 1H, *J*=7.9, H-11); LC-MS, *m/z* = 316 [M+1], 318 [M+3]; Anal. calcd. for C_15_H_17_N_5_OS: C, 57.12; H, 5.43; N, 22.20; S, 10.17; Found: C, 57.13; H, 5.43; N, 22.21; S, 10.18.

###### 6-{[2-(Dimethylamino)ethyl]thio}-3-phenyl-2H-[1,2,4]triazino[2,3-c]quinazolin-2-one (**3.12**)

Yield: Method B, 92.7%, M.p. 104–106°C; IR (cm^−1^): 3058, 2974, 2916, 2849, 2812, 2757, 2723, 1669, 1658, 1582, 1555, 1503, 1487, 1464, 1456, 1444, 1373, 1337, 1313, 1295, 1282, 1264, 1236, 1212, 1180, 1160, 1135, 1101, 1078, 1056, 1042, 1019, 1000, 986, 961, 939, 901, 876, 849, 811, 767, 750, 688, 650, 621; ^1^H-NMR (500 MHz, DMSO-d6, TMS): δ=2.26 (s, 6H, -N(C*H*_3_)_2_, 2.67 (t, 2H, *J*=6.9, -S-CH*_2_*-C*H*_2_-), 3.41 (t, 2H, *J*=6.9, -S-C*H**_2_*-), 7.64–7.59 (m, 3H, H-3′,4′,5′ Ph), 7.67 (t, 1H, *J*=7.3, H-10), 7.74 (d, 1H, *J*=7.9, H-8), 7.96 (t, 1H, *J*=7.3, H-9), 8.26 (d, 2H, *J*=7.3, H-2′,6′ Ph), 8.42 (d, 1H, *J*=7.9, H-11); ^13^C-NMR (100 MHz): δ=29.44 (-S*C*H_2_), 45.38 ((*C*H_3_)_2_N-), 57.64 (-CH_2_-N-), 118.13 (11a), 126.03 (8), 126.82 (10), 127.87 (11), 128.85 (3′, 5′ Ph), 129.77 (2′, 6′ Ph), 131.93 (4′ Ph), 132.15 (1′ Ph), 136.03 (9), 144.24 (3), 149.57 (11b), 151.03 (7a), 155.58 (6), 160.01 (2); EI-MS, *m/z* (I_rel_, %) = 170 (5.8), 103 (27.3), 102 (13.0), 90 (7.5), 77 (5.0), 76 (17.1), 72 (68.2), 71 (36.8), 70 (28.9), 63 (8.2), 59 (22.8), 58 (100.0), 56 (30.7), 44 (7.8), 43 (18.1), 42 (23.7); Anal. calcd. for C_20_H_19_N_5_OS: C, 63.64; H, 5.07; N, 18.55; S, 8.49; Found: C, 63.66; H, 5.07; N, 18.56; S, 8.51.

###### 6-{[2-(Dimethylamino)ethyl]thio}-3-(4-methylphenyl)-2H-[1,2,4]triazino[2,3-c]quinazolin-2-one (**3.13**)

Yield: Method B, 51.0%, M.p. 130–133°C; IR (cm^−1^): 2942, 2917, 2849, 2806, 2776, 2756, 2728, 1664, 1608, 1584, 1561, 1547, 1513, 1495, 1467, 1372, 1338, 1319, 1308, 1281, 1267, 1239, 1184, 1161, 1135, 1105, 1068, 1042, 1018, 985, 962, 939, 898, 830, 769, 712, 700, 684, 625; ^1^H-NMR (500 MHz, DMSO-d6, TMS): δ=2.28 (s, 6H, -N(C*H*_3_)_2_, 2.40 (s, 3H, C*H*_3_), 2.69 (t, 2H, *J*=6.9, -S-CH*_2_*-C*H*_2_-), 3.39 (t, 2H, *J*=6.9, -S-C*H**_2_*-), 7.38 (d, 2H, *J*=7.7, H-3,′4′ Ph), 7.65 (t, 1H, *J*=7.3, H-10), 7.72 (d, 1H, *J*=7.9, H=8), 7.94 (t, 1H, *J*=7.3, H-9), 8.20 (d, 2H, *J*=7.7, H-2′,6′ Ph), 8.45 (d, 1H, *J*=7.9, H-11); LC-MS, *m/z* = 392 [M+1], 394 [M+3]; Anal. calcd. for C_21_H_21_N_5_OS: C, 64.43; H, 5.41; N, 17.89; S, 8.19; Found: C, 64.43; H, 5.42; N, 17.81; S, 8.21.

###### 6-{[2-(Dimethylamino)ethyl]thio}-3-(4-methoxyphenyl)-2H-[1,2,4]triazino[2,3-c]quinazolin-2-one (**3.14**)

Yield: Method B, 68.7%, M.p. 158–162°C; IR (cm^−1^): 3013, 2969, 2941, 2824, 2777, 2705, 1666, 1603, 1580, 1557, 1538, 1513, 1494, 1465, 1418, 1368, 1337, 1318, 1304, 1264, 1234, 1171, 1138, 1103, 1066, 1051, 1018, 985, 952, 937, 889, 831, 799, 769, 722, 702, 683, 668, 640, 621; ^1^H-NMR (500 MHz, DMSO-d6, TMS): δ=2.32 (s, 6H, -N(C*H*_3_)_2_, 2.74 (t, 2H, -S-CH*_2_*-C*H*_2_-), 3.43 (t, 2H, -S-C*H**_2_*-), 3.87 (s, 3H, CH_3_O), 7.11 (d, 2H, H-3,′4′ Ph), 7.77–7.60 (m, 2H, H-8, 10), 7.93 (t, 1H, H-9), 8.35 (d, 2H, H-2′,6′ Ph), 8.47 (d, 1H, H-11); LC-MS, *m/z* = 408 [M+1], 410 [M+3]; Anal. calcd. for C_21_H_21_N_5_O_2_S: C, 61.90; H, 5.19; N, 17.19; S, 7.87; Found: C, 61.91; H, 5.19; N, 17.20; S, 7.88.

###### 6-{[2-(Diethylamino)ethyl]thio}-3-methyl-2H-[1,2,4]triazino[2,3-c]quinazolin-2-one (**3.15**)

Yield: Method B, 58.2%, M.p. 120–122°C; IR (cm^−1^): 2968, 2919, 2806, 1663, 1624, 1600, 1580, 1555, 1502, 1463, 1425, 1374, 1359, 1340, 1312, 1282, 1258, 1218, 1208, 1193, 1132, 1069, 1042, 1027, 952, 906, 882, 859, 764, 739, 728, 700, 685, 663, 630, 607; ^1^H-NMR (500 MHz, DMSO-d6, TMS): δ=1.04 (t, 6H, *J*=6.9, -N(CH_2_-C*H*_3_)_2_, 2.36 (s, 3H, CH_3_), 2.61 (qui, 4H, *J^2^*=13.9, *J^3^*=6.2, -N(C*H*_2_-CH_3_)_2_), 2.81 (t, 2H, *J*=6.9, -S-CH*_2_*-C*H*_2_-), 3.31 (t, 2H, *J*=6.9, -S-C*H**_2_*-), 7.70–7.61 (m, 2H, H-8, 10), 7.95 (t, 1H, *J*=7.1, H-9), 8.43 (d, 1H, *J*=7.9, H-11); LC-MS, *m/z* = 344 [M+1], 346 [M+3]; Anal. calcd. for C_17_H_21_N_5_OS: C, 59.45; H, 6.16; N, 20.39; S, 9.34; Found: C, 59.45; H, 6.16; N, 20.40; S, 9.36.

###### 6-{[2-(Diethylamino)ethyl]thio}-3-phenyl-2H-[1,2,4]triazino[2,3-c]quinazolin-2-one (**3.16**)

Yield: Method B, 67.8%, M.p. 118–120°C; IR (cm^−1^): 3058, 2963, 2926, 2868, 2813, 1659, 1587, 1556, 1505, 1488, 1469, 1445, 1402, 1372, 1339, 1317, 1284, 1269, 1245, 1206, 1188, 1164, 1138, 1106, 1070, 1021, 1002, 987, 940, 851, 813, 778, 768, 754, 689, 652, 623, 613; ^1^H-NMR (500 MHz, DMSO-d6, TMS): δ=1.05 (t, 6H, *J*=7.1, -N(CH_2_-C*H*_3_)_2_ 2.62 (qui, 4H, *J^2^*=14.3, *J^3^*=7.1, -N(C*H*_2_-CH_3_)_2_), 2.83 (t, 2H, *J*=7.1, -S-CH*_2_*-C*H*_2_-), 3.34 (t, 2H, *J*=7.1, -S-C*H**_2_*-), 7.63–7.56 (m, 3H, H-3′,4′, 5′ Ph), 7.67 (t, 1H, *J^3^*=7.5, H-10), 7.73 (d, 1H, *J*=7.9, H-8), 7.98 (t, 1H, *J^3^*=7.1, *J^4^*=1.6, H-9), 8.27 (d, 2H, *J*=6.9, H-2′, 6′ Ph), 8.48 (d, 1H, *J*=7.9, H-11); ^13^C-NMR (100 MHz): δ=12.56 ((*C*H_3_CH_2_)_2_N-), 28.89 (-S*C*H_2_), 46.91 ((CH_3_*C*H_2_)_2_N-), 51.36 (-CH_2_-N-), 118.27 (11a), 126.14 (8), 126.71 (10), 127.90 (11), 128.91 (3′, 5′ Ph), 129.79 (2′, 6′ Ph), 131.97 (4′ Ph), 132.22 (1′ Ph), 136.08 (9), 144.34 (3), 149.54 (11b), 151.14 (7a), 155.55 (6), 160.10 (2); LC-MS, *m/z* = 406 [M+1], 408 [M+3]; EI-MS, *m/z* (I_rel_, %) = 103 (18,5), 102 (6,0), 100 (17,2), 99 (100,0), 86 (13,1), 76 (9,9), 72 (6,2), 71 (35,0), 70 (13,5),44 (6,9), 42 (9,1); Anal. calcd. for C_22_H_23_N_5_OS: C, 65.16; H, 5.72; N, 17.27; S, 7.91; Found: C, 65.14; H, 5.72; N, 17.25; S, 7.89.

###### 6-{[2-(Diethylamino)ethyl]thio}-3-(4-methylphenyl)-2H-[1,2,4]triazino[2,3-c]quinazolin-2-one (**3.17**)

Yield: Method B, 84.0%, M.p. 97–99°C; IR (cm^−1^): 3061, 3035, 2962, 2918, 2825, 2784, 1658, 1611, 1588, 1562, 1546, 1497, 1469, 1371, 1339, 1322, 1305, 1268, 1239, 1204, 1182, 1162, 1136, 1107, 1084, 1067, 1035, 1020, 989, 960, 940, 875, 853, 831, 808, 771, 720, 704, 683, 641, 626; ^1^H-NMR (500 MHz, DMSO-d6, TMS): δ=1.05 (t, 6H, *J*=7.1, -N(CH_2_-C*H*_3_)_2_ 2.40 (s, 3H, CH_3_), 2.62 (qui, 4H, *J^2^*=14.3, *J^3^*=7.1, -N(C*H*_2_-CH_3_)_2_), 2.83 (t, 2H, *J*=6.9, -S-CH*_2_*-C*H*_2_-), 3.32 (t, 2H, *J*=6.9, -S-C*H**_2_*-), 7.37 (d, 2H, *J*=7.7, H-3′,4′ Ph), 7.70–7.63 (m, 2H, H-8, 10), 7.95 (t, 1H, *J^3^*=7.3, H-9), 8.21 (d, 2H, *J*=7.7, H-2′, 6′ Ph), 8.45 (d, 1H, *J*=7.9, H-11); LC-MS, *m/z* = 420 [M+1], 422 [M+3]; Anal. calcd. for C_23_H_25_N_5_OS: C, 65.85; H, 6.01; N, 16.69; S, 7.64; Found: C, 65.85; H, 6.01; N, 16.69; S, 7.64.

###### 6-{[2-(Diethylamino)ethyl]thio}-3-(4-methoxyphenyl)-2H-[1,2,4]triazino[2,3-c]quinazolin-2-one (**3.18**)

Yield: Method B, 68.8%, M.p. 130–132°C; IR (cm^−1^): 2967, 2931, 2873, 2836, 2801, 1662, 1604, 1586, 1562, 1545, 1513, 1495, 1463, 1421, 1371, 1340, 1320, 1305, 1286, 1271, 1255, 1239, 1175, 1138, 1108, 1069, 1018, 988, 940, 838, 810, 798, 769, 722, 702, 684, 637, 624; ^1^H-NMR (500 MHz, DMSO-d6, TMS): δ=1.05 (t, 6H, *J*=7.1, -N(CH_2_-C*H*_3_)_2_), 2.61 (qui, 4H, *J^2^*=14.3, *J^3^*=7.1, -N(C*H*_2_-CH_3_)_2_), 2.82 (t, 2H, *J*=6.9, -S-CH*_2_*-C*H*_2_-), 3.32 (t, 2H, *J*=6.9, -S-C*H**_2_*-), 3.86 (c, 3H, CH_3_O), 7.12 (d, 2H, *J*=8.7, H-3′,4′ Ph), 7.71–7.62 (m, 2H, H-8, 10), 7.95 (t, 1H, *J^3^*=7.3, H-9), 8.34 (d, 2H, *J*=8.7, H-2′, 6′ Ph), 8.44 (d, 1H, *J*=7.9, H-11); Anal. calcd. for C_23_H_25_N_5_O_2_S: C, 63.43; H, 5.79; N, 16.68; S, 7.36; Found: C, 63.43; H, 5.79; N, 16.68; S, 7.36.

###### 6-{[2-(Diisopropylamino)ethyl]thio}3-methyl-2H-[1,2,4]triazino[2,3-c]quinazolin-2-one (**3.19**)

Yield: Method B, 56.5%, M.p. 96–98°C; IR (cm^−1^): 2960, 2921, 2870, 2815, 1664, 1606, 1581, 1556, 1504, 1465, 1432, 1391, 1363, 1337, 1307, 1284, 1260, 1206, 1158, 1132, 1116, 1036, 953, 885, 859, 770, 695, 685, 628; ^1^H-NMR (500 MHz, DMSO-d6, TMS): δ=1.05 (d, 12H, *J*=6.4, -N((CH-(C*H*_3_)_2_)_2_), 2.35 (s, 3H, CH_3_), 2.78 (t, 2H, *J*=6.9, -S-CH*_2_*-C*H*_2_-), 3.09 (qui, 2H, *J^2^*=12.5, *J^3^*=6.4, -N((C*H*-(CH_3_)_2_)_2_, 3.20 (t, 2H, *J*=6.9, -S-C*H**_2_*-), 7.76–7.61 (m, 2H, H-8, 10), 7.94 (t, 1H, *J*=7.1, H-9), 8.42 (d, 1H, *J*=7.9, H-11); LC-MS, *m/z* = 372 [M+1], 374 [M+3]; Anal. calcd. for C_19_H_25_N_5_OS: C, 61.43; H, 6.78; N, 18.85; S, 8.63; Found: C, 61.40; H, 6.78; N, 18.83; S, 8.60.

###### 6-{[2-(Diisopropylamino)ethyl]thio}3-phenyl-2H-[1,2,4]triazino[2,3-c]quinazolin-2-one (**3.20**)

Yield: Method B, 80.7%, M.p. 124–126°C; IR (cm^−1^): 3094, 3053, 2964, 2925, 2870, 2777, 1659, 1591, 1554, 1503, 1485, 1470, 1401, 1378, 1339, 1321, 1311, 1284, 1262, 1238, 1202, 1184, 1158, 1139, 1114, 1086, 1048, 1029, 1002, 986, 963, 939, 889, 871, 850, 811, 781, 773, 750, 727, 703, 688, 652, 614; ^1^H-NMR (500 MHz, DMSO-d6, TMS): δ=1.05 (d, 12H, *J*=6.4, -N((CH-(C*H*_3_)_2_)_2_), 2.82 (t, 2H, *J*=6.9, -S-CH*_2_*-C*H*_2_-), 3.11 (qui, 2H, *J^2^*=12.5, *J^3^*=6.4, -N((C*H*-(CH_3_)_2_)_2_, 3.26 (t, 2H, *J*=6.9, -S-C*H**_2_*-), 7.64–7.56 (m, 3H, H-3′,4′, 5′ Ph), 7.73–7.67 (m, 2H, H-8, 10), 7.98 (t, 1H, *J^3^*=7.1, *J^4^*=1.6, H-9), 8.29 (d, 2H, *J*=6.9, H-2′, 6′ Ph), 8.49 (d, 1H, *J*=7.9, H-11); ^13^C-NMR (100 MHz): δ=21.42 ((*C*H_3_CH_2_CH_2_)_2_N-), 32.31 (-SCH_2_), 44.51 ((CH_3_*C*H_2_CH_2_)_2_N-), 48.84 (-CH_2_-N-), 66.86 ((CH_3_CH_2_*C*H_2_)_2_N-), 118.33 (11a), 126.16 (8), 126.58 (10), 127.88 (11), 128.90 (3′, 5′ Ph), 129.77 (2′, 6′ Ph), 132.00 (4′ Ph), 132.21 (1′ Ph), 136.10 (9), 144.37 (3), 149.42 (11b), 151.10 (7a), 159.49 (6), 160.08 (2); LC-MS, *m/z* = 434 [M+1], 436 [M+3]; Anal. calcd. for C_24_H_27_N_5_OS: C, 66.49; H, 6.28; N, 16.15; S, 7.40; Found: C, 66.50; H, 6.29; N, 16.16; S, 7.41.

###### 6-{[2-(Diisopropylamino)ethyl]thio}3-(4-methylphenyl)-2H-[1,2,4]triazino[2,3-c]quinazolin-2-one (**3.21**)

Yield: Method B, 53.0%, M.p. 141–144°C; IR (cm^−1^): 2958, 2925, 2870, 1665, 1609, 1589, 1562, 1547, 1498, 1467, 1407, 1394, 1382, 1359, 1339, 1320, 1306, 1279, 1266, 1237, 1202, 1183, 1158, 1133, 1106, 1070, 1051, 1038,1021, 984, 962, 938, 884, 867, 854, 833, 771, 756, 712, 699, 685, 679, 640, 625, 603; ^1^H-NMR (500 MHz, DMSO-d6, TMS): δ=1.07 (d, 12H, *J*=6.0, -N((CH-(C*H*_3_)_2_)_2_), 2.41 (s, 3H, CH_3_), 2.82 (t, 2H, *J*=6.9, -S-CH*_2_*-C*H*_2_-), 3.11 (qui, 2H, N((C*H*-(CH_3_)_2_)_2_), 3.25 (t, 2H, *J*=6.9, -S-C*H**_2_*-), 7.37 (d, 2H, *J*=7.7, H-3′,4′ Ph), 7.70–7.64 (m, 2H, H-8, 10), 7.97 (t, 1H, *J*=7.3, H-9), 8.23 (d, 2H, *J*=7.7, H-2′, 6′ Ph), 8.47 (d, 1H, *J*=7.9, H-11); EI-MS, *m/z* (I_rel_, %) = 438 (2.0), 230 (7.7), 161 (13.1), 134 (5.6), 133 (5.7), 131 (6.6), 130 (8.8), 129 (18.0), 128 (17.6), 127 (88.8), 104 (7.4), 103 (13.4), 102 (23.9), 99 (16.6), 98 (29.5), 97 (23.9), 96 (6.3), 95 (9.7), 75 (6.9), 73 (11.1), 72 (70.8), 71 (49.8), 70 (96.7), 69 (67.5), 68 (19.5), 67 (21.8), 65 (11.6), 64 (8.3), 63 (10.3), 44 (20.2), 43 (100.0), 42 (38.2), 41 (93.4), 40 (24.4); LC-MS, *m/z* = 448 [M+1], 450 [M+3]; Anal. calcd. for C_25_H_29_N_5_OS: C, 67.09; H, 6.53; N, 15.65; S, 7.16; Found: C, 67.06; H, 6.53; N, 15.63; S, 7.14.

###### 6-{[2-(Diisopropylamino)ethyl]thio}3-(4-methoxyphenyl)-2H-[1,2,4]triazino[2,3-c]quinazolin-2-one **(3.22**)

Yield: Method B, 75.5%, M.p. 148–150°C; IR (cm^−1^): 3014, 2959, 2929, 2866, 1665, 1603, 1588, 1563, 1546, 1515, 1500, 1465, 1428, 1392, 1358, 1340, 1319, 1301, 1283, 1270, 1257, 1238, 1206, 1170, 1157, 1133, 1112, 1051, 1018, 1011, 985, 963, 938, 884, 838, 810, 799, 754, 723, 699, 681, 634, 623; ^1^H-NMR (500 MHz, DMSO-d6, TMS): δ=1.08 (d, 12H, *J*=6.0, -N((CH-(C*H*_3_)_2_)_2_), 2.83 (t, 2H, *J*=6.9, -S-CH*_2_*-C*H*_2_-), 3.12 (qui, 2H, N((C*H*-(CH_3_)_2_)_2_), 3.25 (t, 2H, *J*=6.9, -S-C*H**_2_*-), 3.86 (s, 3H, CH_3_O), 7.12 (d, 2H, *J*=8.7, H-3′,4′ Ph), 7.72–7.64 (m, 2H, H-8, 10), 7.96 (t, 1H, *J*=7.7, H-9), 8.37 (d, 2H, *J*=8.7, H-2′, 6′ Ph), 8.47 (d, 1H, *J*=7.9, H-11); LC-MS, *m/z* = 464 [M+1], 466 [M+3]; Anal. calcd. for C_25_H_29_N_5_O_2_S: C, 63.77; H, 6.31; N, 15.11; S, 6.92; Found: C, 63.77; H, 6.31; N, 15.11; S, 6.92.

### Pharmacology

#### Bioluminescence inhibition test

The marine luminescent bacteria *Photobacterium leiognathi* strain Sh1, isolated from the Azov Sea Shrimp, were used for the bioluminescence analysis [33]. Bacteria were cultivated in a nutrient environment containing (g/L): pepton – 5, yeast extract – 1.5, meat extract – 1.5, sodium chloride – 30, pH = 7.4. In the acute action test (inhibiting luminescence of bacteria), bacteria were diluted with the 3% sodium chloride solution down to a concentration of 10^5^ cells/mL. 5–50 mg/mL of the studied substances suspended in DMSO were mixed with 1 mL of the diluted bacterial suspension. Vials were incubated for 10 min at 25°C, then, the intensity of bioluminescence was measured in percent (%) relative to the controls, which were prepared without the studied compounds. In the chronic action test (inhibiting growth and luminescence of bacteria), growth medium was added for potential breeding in a ratio of 1:50 and the mix was incubated for 16–18 h at 30°C, whereupon the intensity of bioluminescence was measured the same way as in acute action testing. Tetracycline was used as a reference. The bacterial luminescence was measured with a Bioluminometer BLM-8801 («Science», Krasnoyarsk, Russia).

#### Antimicrobial and antifungal test

The investigation of antimicrobial and antifungal activity of compounds **2.1–2.8** and **3.1–3.22** was carried out with the stiff plate agar diffusion method against *Escherichia coli*, *Staphylococcus aureus*, *Mycobacterium luteum*, *Candida tenuis* and *Aspergillus niger*. The amount of microbial cells was 109 c.f.u./mL. Incubation period of bacteria was 24 h at 35°C, yeast − 48 to 72 h at 28–30°C. Antibiotics vancomicin, oxacillin, nystatin were used as standards. The bacterial cultures, standards and the obtained substances were streaked across grooves at 5 mg/mL concentration, and then allowed to diffuse in the agar nutrient plate. The antimicrobial effect and degree of activity of the tested compounds were evaluated by measuring of the inhibition zone diameters (low sensitive: 11–15 mm; sensitive: 16–25 mm; highly sensitive >25 mm). All experiments were repeated three times.

#### Cytotoxic activity against malignant human tumor cells

Primary anticancer assay was performed at human tumor cell lines panel derived from nine neoplastic diseases, in accordance with the protocol of the Drug Evaluation Branch, National Cancer Institute, Bethesda [24–29]. Tested compounds were added to the culture at a single concentration (10^−5^ M) and the cultures were incubated for 48 h. End point determinations were made with a protein binding dye, sulforhodamine B (SRB). Results for each tested compound were reported as the percent of growth of the treated cells when compared to the untreated control cells. The percentage growth was evaluated spectrophotometrically versus controls not treated with test agents. The cytotoxic and/or growth inhibitory effects of the most active selected compounds were tested *in vitro* against the full panel of about 60 human tumor cell lines at 10-fold dilutions of five concentrations ranging from 10^−4^ to 10^−8^ M. A 48-h continuous drug exposure protocol was followed and an SRB protein assay was used to estimate cell viability or growth. Using the seven absorbance measurements [time zero, (T_z_), control growth in the absence of drug (C), and test growth in the presence of drug at the five concentration levels (T_i_)], the percentage growth was calculated at each of the drug concentrations levels. Percentage growth inhibition was calculated as:

$$\frac{{\text{T}}_{\text{i}}-{\text{T}}_{\text{z}}}{\text{C}-{\text{T}}_{\text{z}}}\times 100$$

for concentrations for which T_i_ ≥ T_z_,

$$\frac{{\text{T}}_{\text{i}}-{\text{T}}_{\text{z}}}{{\text{T}}_{\text{z}}}\times 100$$

for concentrations for which T_i_ < T_z_.

Three dose response parameters were calculated for each compound. Growth inhibition of 50% (GI_50_) was calculated from [(T_i_ − T^z^)/(C − T_z_)] × 100 = 50, which is the drug concentration resulting in a 50% lower net protein increase in the treated cells (measured by SRB staining) as compared to the net protein increase seen in the control cells. The drug concentration resulting in total growth inhibition (TGI) was calculated from T_i_ = T_z_. The LC_50_ (concentration of drug resulting in a 50% reduction in the measured protein at the end of the drug treatment as compared to that at the beginning) indicating a net loss of cells following treatment was calculated from [(T_i_ − T_z_)/T_z_] ×100 = −50. Values were calculated for each of these three parameters if the level of activity was reached; however, if the effect was not reached or was exceeded, the value for that parameter was expressed as greater or less than the maximum or minimum concentration tested. The log GI_50_, log TGI, log LC_50_ were then determined, defined as the mean of the log’s of the individual GI_50_, TGI, LC_50_ values. The lowest values were obtained

## Conclusion

In the present paper, 22 new 6-{[ω-(dialkylamino(heterocyclyl)alkyl]thio}-3-R-2*H*-[1,2,4]triazino[2,3-*c*]quinazoline-2-ones were described. Six of the synthesized compounds were tested for anticancer activity and most of them inhibited the growth of the leukemia, melanoma, lung, colon, CNS, ovarian, renal, prostate and breast cancers cell lines. In conclusion, these preliminary results allowed identification of the most active compounds **3.14**, **3.16** and **3.18**. The latter 6-{[2-(diethylamino)ethyl]thio}-3-(4-methoxyphenyl)-2*H*-[1,2,4]triazino[2,3-*c*]quinazolin-2-one (**3.18**) could be prospective antitumor agent (log GI_50_=−5,69 and log TGI=−5.14) with the selective influence on the colon cancer cell lines. The obtained results prove the necessity for further investigations to clarify the features underlying the antitumor potential of the tested compounds.

## Figures and Tables

**Sch. 1:**
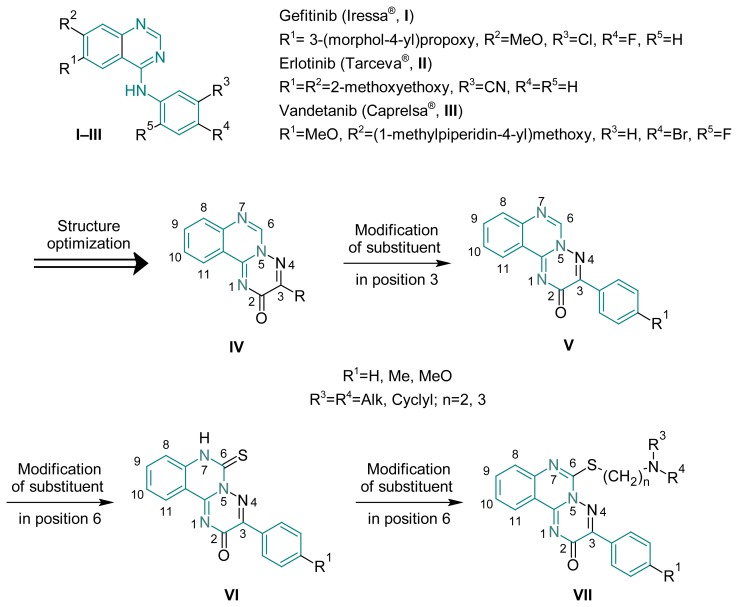
Structures of quinazoline-based compounds and their medicinal chemistry optimization.

**Sch. 2:**
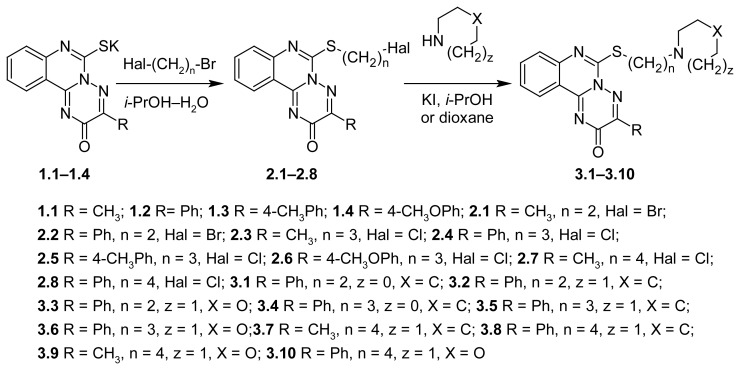
Structures of quinazoline-based compounds and their medicinal chemistry optimization.

**Sch. 3:**
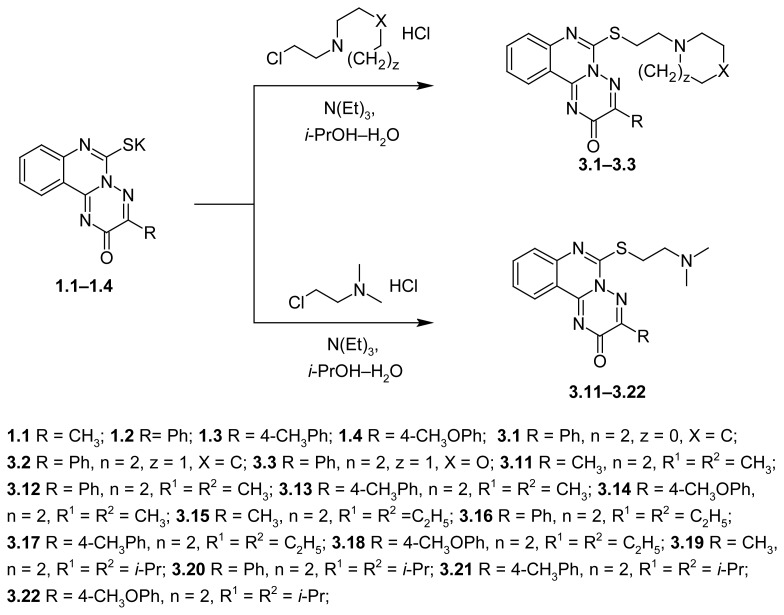
Synthesis of the 6-{[2-(heterocyclyl)ethyl]thio}-3-R-2*H*-[1,2,4]triazino[2,3-c]quinazoline-2-one (**3.1–3.3**) and 6-{[2-(dialkylamino)ethyl]thio}-3-R-2*H*-[1,2,4]triazino[2,3-c]quinazoline-2-ones (**3.11–3.22**).

**Tab. 1:** Bioluminescence intensity, %

Compd.	Control	Acute action test, mg/mL			Chronic action test, mg/mL		

		0.025	0.1	0.25	0.025	0.1	0.25
DMSO	100.0	100.00	100.00	100.00	100.00	100.00	100.00
2.1	100.0	111.50	99.10	122.60	150.00	80.00	73.30
2.2	100.0	98.85	94.25	2.30	100.00	53.33	18.33
2.3	100.0	81.50	74.50	73.08	122.50	92.50	78.00
2.4	100.0	52.31	53.85	38.30	72.00	64.00	28.00
2.5	100.0	100.00	101.02	97.96	16.67	40.67	30.04
2.6	100.0	75.00	89.29	62.50	150.94	65.94	27.55
2.7	100.0	102.20	125.20	79.10	66.00	63.60	85.50
2.8	100.0	125.00	100.00	93.30	64.80	52.00	57.10
3.1	100.0	67.16	14.93	2.99	0.36	0.00	0.00
3.2	100.0	64.62	18.46	4.62	0.00	0.00	0.00
3.3	100.0	23.08	5.77	3.85	0.33	0.00	0.00
3.4	100.0	81.50	90.00	115.20	93.30	166.40	80.0
3.5	100.0	124.00	120.00	90.00	90.00	90.00	30.00
3.6	100.0	35.56	39.56	49.67	77.24	84.32	32.21
3.7	100.0	125.00	112.50	100.00	84.90	93.60	78.20
3.8	100.0	93.30	65.50	42.50	39.20	63.30	85.00
3.9	100.0	100.00	106.0	128.60	104.4	127.7	87.50
3.10	100.0	100.00	100.9	112.90	57.50	75.60	93.30
3.11	100.0	105.00	30.00	0.00	60.00	29.33	0.00
3.12	100.0	97.96	40.82	10.20	0.00	0.00	0.00
3.13	100.0	49.20	26.50	9.70	0.00	0.00	0.00
3.14	100.0	33.30	4.00	7.50	0.00	0.00	0.00
3.15	100.0	91.80	17.70	0.00	100.0	0.00	0.00
3.16	100.0	10.0	8.00	2.90	0.00	0.00	0.00
3.17	100.0	60.32	34.92	0.00	1.25	0.00	0.00
3.18	100.0	83.30	40.00	12.50	30.2	33.3	75.00
3.19	100.0	100.00	27.91	0.00	58.46	15.38	0.00
3.20	100.0	64.44	88.89	0.00	0.00	0.00	0.00
3.21	100.0	100.00	67.91	0.00	48.26	25.64	0.00
3.22	100.0	89.13	76.09	76.09	56.67	46.26	26.67
Tetracycline	100.0	80.70	9.10	0.00	0.00	0.00	0.00

**Tab. 2:** Antimicrobial activity of synthesized compounds

Compd.^a^	mg/mL	The inhibitory zones of the investigated compounds, mm				

		*E. coli*	*S. aureus*	*M. luteum*	*C. tenuis*	*A. niger*
3.1	5.0	0	0	25	0	0
3.1	1.0	0	0	10	0	0
3.2	5.0	0	0	23	0	0
3.2	1.0	0	0	18	0	0
3.3	5.0	0	0	20	0	0
3.3	1.0	0	0	16	0	0
3.4	5.0	0	0	12	0	0
3.5	5.0	0	0	10	0	0
3.6	5.0	0	0	8	0	0
3.7	5.0	0	0	16	0	0
3.8	5.0	0	0	15	0	0
3.9	5.0	0	0	12	0	0
3.10	5.0	0	0	13	0	0
3.11	5.0	0	0	8	0	0
3.12	5.0	0	12	26	0	0
3.12	1.0	0	0	14	0	0
3.13	5.0	0	9	23	0	6^b^
3.13	1.0	0	0	16	0	0
3.14	5.0	0	0	9	0	0
3.15	5.0	0	0	12	0	0
3.16	5.0	0	8	24	0	0
3.16	1.0	0	0	12	0	0
3.17	5.0	0	12	20	0	12^b^
3.17	1.0	0	0	7	0	0
3.18	5.0	0	13	21	8	23^b^
3.18	1.0	0	7	15	0	0
3.19	5.0	0	0	7	0	0
3.20	5.0	0	0	28	0	0
3.20	1.0	0	0	17	0	0
3.21	5.0	0	0	12	0	0
3.21	1.0	0	0	8	0	0
3.22	5.0	0	0	7	0	0
Vancomicin	0.1	16	18	58	0	0
Nystatin	0.1	0	11	15	24	25
Oxacillin	0.1	0	21	0	0	0

a compounds **2.1–2.8** at concentration of 1.0 and 5.0 mg/mL didn’t inhibit investigated bacteria;

b zone of late spore formation of *Aspergillus niger* (without inhibition of *fungus mycelium*).

**Tab. 3:** Cytotoxic activity of the compounds in conc. 10^−5^ M against 60 cell cancer lines

Cpd.	Mean growth, %	Range of growth, %	Most sensitive cell line growth, %^a^
3.1	98.17	−39.37–137.18	−39.37 (CCRF-CEM/L), −8.71 (HL-60(TB)/L), 56.76 (SR/L)
3.14	64.02	−9.70–111.63	45.39 (CCRF-CEM/L), 38.21 (HL-60(TB)/L), 2.60 (K- 562/L), 3.07 (MOLT-4/L), 61.63 (RPMI-8226/L), −9.70 (SR/L), 32.00 (A549/ATCC/nscLC), 24.43 (NCI- H460/nscLC), 41.37 (NCI-H522/nscLC), 32.77 (HCT- 116/ColC), 27.77 (HT29/ColC), 38.36 (KM12/ColC), 32.21 (SW-620/ColC), 58.33 (SF-268/CNSC), 54.01 (SF-539/CNSC), 43.12 (LOX IMVI/M), 56.09 (OVCAR- 3/OV), 38.05 (OVCAR-8/OV), 32.25 (ACHN/RC)
3.15	102.73	47.89–120.73	51.70 (K-562/L), 47.89 (MOLT-4/L)
3.16	36.73	3.14–86.74	27.70 (CCRF-CEM/L), 14.32 (K-562/L), 14.03 (MOLT- 4/L), 22.57 (RPMI-8226/L), 3.14 (SR/L), 16.17 (A549/ATCC/nscLC), 50.95 (EKVX/nscLC), 39.76 (HOP-62/nscLC), 50.31 (NCI-H226/nscLC), 56.43 (NCI-H23/nscLC), 14.76 (NCI-H460/nscLC), 5.40 (NCI-H522/nscLC), 20.35 (COLO 205/ColC), 51.28 (HCC-2998/ColC), 27.21 (HCT-116/ColC), 30.10 (HCT-15/ColC), 5.98 (HT29/ColC), 32.28 (KM12/ColC), 27.71 (SW-620/ColC), 49.83 (SF- 268/CNSC), 36.94 (SF-295/CNSC), 36.17 (SF- 539/CNSC), 44.31 (SNB-75/CNSC), 27.70 (U251/CNSC), 25.46 (LOX IMVI/M), 36.43 (MDA-MB- 435/M), 23.34 (SK-MEL-2/M), 37.44 (SK-MEL-5/M), 3.33 (UACC-257/M), 46.99 (UACC-62/M), 45.11 (OVCAR-3/OV), 49.28 (OVCAR-4/OV), 23.91 (OVCAR-8/OV), 41.06 (NCI/ADR-RES/OV), 49.73 (SK-OV-3/OV), 39.89 (786-0/RC), 45.41 (A498/RC), 24.31 (ACHN/RC), 41.64 (RXF 393/RC), 41.33 (SN12C/RC), 24.20 (TK-10/RC), 47.06 (PC-3/PC), 26.87 (DU-145/PC), 14.90 (MCF7/BC), 37.92 (T- 47D/BC), 36.10 (MDA-MB-468/BC)
3.18	46.48	−86.89–108.61	5.24 (K-562/L), 20.57 (MOLT-4/L), 37.58 (RPMI- 8226/L), −17.24 (SR/L), −38.73 (A549/ATCC/nscLC), 48.66 (HOP-62/nscLC), 40.55 (HOP-92/nscLC), 36.24 (NCI-H322M/nscLC), −86.89 (NCI-H460/nscLC), 36.93 (COLO 205/ColC), 39.88 (HCT-116/ColC), 33.86 (HCT-15/ColC), 3.67 (HT29/ColC), 32.42 (KM12/ColC), 12.48 (SW-620/ColC), 24.34 (SF- 539/CNSC), 24.54 (U251/CNSC), 10.18 (LOX IMVI/M), 40.06 (MALME-3M/M), 25.90 (IGROV1/OV), 47.44 (OVCAR-3/OV), 42.39 (OVCAR-4/OV), 31.58 (OVCAR-8/OV), 56.28 (NCI/ADR-RES/OV), 32.69 (ACHN/RC), 55.08 (RXF 393/RC), 46.16 (SN12C/RC), 10.63 (UO-31/RC), 55.51 (PC-3/PC), 35.97 (DU- 145/PC), 27.57 (MCF7/BC), 56.58 (MDA-MB- 231/ATCC/BC)
3.21	85.86	−36.39–128.76	52.74 (HL-60(TB)/L), −36.39 (K-562/L), −25.77 (SR/L), 53.52 (NCI-H460/nscLC), 22.86 (HCT-116/ColC), 7.75 (SW-620/ColC), 10.81 (MDA-MB-435/M)

L…leukemia; nscLC…non-small cell lung cancer; ColC…colon cancer; CNSC…CNS cancer; M…melanoma; OV…ovarian cancer; RC…renal cancer; PC…prostate cancer; BC…breast cancer.

**Tab. 4:** Summary of anticancer screening data at dose-dependent assay

Comp.	*N*^a^	log GI_50_			log TGI			log LC_50_		

		*N1*^b^	Range	MG_MID	*N2*^b^	Range	MG_MID	*N3*^b^	Range	MG_MID
3.14	57	−55	−6.07 to −5.01	−5.48	17	−5.53 to −4.02	−4.21	5	−5.46 to −4.04	−4.05
3.16	59	59	−6.29 to −5.31	−5.52	58	−5.44 to −4.64	−4.89	50	−5.11 to −4.07	−4.32
3.18	59	59	−6.29 to −5.31	−5.57	59	−5.54 to −4.55	−5.04	56	−5.24 to −4.01	−4.51

a *N* – number of human tumor cell lines tested at the 2nd stage assay;

b *N1, N2, N3* – number of sensitive cell lines, against which the compound possessed considerable growth inhibition according to mentioned parameter (log GI50, log TGI and log LC50 ≤ 4.00).

**Tab. 5:** The influence of compounds **3.14, 3.16** and **3.18** on the growth of individual tumor cell lines (log GI50 ≤ −5.65)

Cpd.	Cancer	Cell line	log GI_50_	log TGI	Log LC_50_
**3.14**	Leukemia	RPMI-8226	−5.70	> −4.00	> −4.00
		SR	−5.83	> −4.00	> −4.00
	NSC lung cancer	A549/ATCC	−5.66	> −4.00	> −4.00
	Colon cancer	HCT-116	−5.66	−4.97	> −4.00
		HT29	−5.74	> −4.00	> −4.00
	CNS cancer	SF-539	−5.80	−5.50	−5.21
		SNB-75	−6.07	−4.90	−4.36
	Melanoma	LOX IMVI	−5.88	−5.53	−5.18
	Renal cancer	A498	−5.88	−4.51	> −4.00
		ACHN	−5.88	−5.46	−5.46
		CAKI-1	−5.94	> −4.00	> −4.00
		UO-31	−5.79	−4.61	> −4.00

**3.16**	Colon cancer	COLO 205	−5.76	−5.37	−4.93
		HT29	−5.66	−4.91	> −4.00
	CNS cancer	SF-539	−5.67	−5.22	−4.62
		SNB-75	−5.72	−4.87	−4.43
	Melanoma	LOX IMVI	−5.76	−5.44	−5.11
		SK-MEL-5	−5.66	−5.23	−4.67
		UACC-257	−5.68	−5.29	−4.77
	Renal cancer	A498	−6.29	−5.21	−4.51
		ACHN	−5.68	−4.93	−4.33
		CAKI-1	−5.66	−4.98	−4.07
		UO-31	−5.79	−5.07	−4.43

**3.18**	Leukemia	RPMI-8226	−5.71	−5.31	−4.51
	NSC lung cancer	A549/ATCC	−5.77	−5.48	−5.20
		HOP-92	−6.20	−5.14	−4.47
		NCI-H460	−5.80	−5.49	−5.19
		NCI-H522	−5.80	−5.49	−5.18
	Colon cancer	COLO 205	−5.88	−5.54	−5.20
		HCT-116	−5.93	−5.42	−4.79
		HT29	−5.96	−5.50	−5.03
		SW-620	−5.65	−4.96	−4.43
	CNS cancer	SF-539	−5.79	−5.45	−5.10
		SNB-75	−5.81	−5.33	−4.70
		U251	−5.78	−5.46	−5.15
	Melanoma	LOX IMVI	−5.89	−5.28	−4.61
		MALME-3M	−5.68	−5.29	−4.65
		SK-MEL-5	−5.74	−5.34	−4.86
		UACC-257	−5.79	−5.51	−5.24
		UACC-62	−5.79	−5.38	−4.79
	Ovarian cancer	OVCAR-3	−5.78	−5.46	−5.15
		OVCAR-4	−5.75	−5.33	−4.82
		OVCAR-8	−5.84	−5.46	−5.08
	Renal cancer	786-0	−5.73	−5.33	−4.84
		ACHN	−5.81	−5.20	−4.45
		RXF 393	−5.75	−5.34	−4.74
	Prostate cancer	PC-3	−5.70	−5.01	−4.47
	Breast cancer	MCF7	−5.81	−5.39	−4.90
		MDA-MB-231/ATCC	−5.76	−5.17	−4.48
		MDA-MB-468	−5.75	−5.37	−4.98

**Tab. 6:** COMPARE analysis of tested compounds^a^

Cpd. No.	PCC	Target	Target vector NSC	Cell lines	Seed StDev	Target StDev	Target mechanism of action^b^
**3.14**	0.601	Maytansine	S153858	57	0.299	0.697	inhibit or promote polymerization of microtubules
	0.556	Vinblastine sulfate	S49842	56	0.300	0.587	inhibit or promote polymerization of microtubules
	0.523	Aclacino- mycin A	S208734	48	0.238	0.135	inhibiting or preventing the proliferation of neoplasms
	0.485	Batracylin	S320846	42	0.239	0.271	inhibitor of DNA topoisomerases I and II induces histone gamma-H2AX
	0.477	Vincristine sulfate	S67574	57	0.299	0.647	inhibit or promote polymerization of microtubules
	0.474	Morpholino- ADR	S354646	57	0.299	0.368	inhibits the progression of the enzyme topoisomerase II
	0.438	*N,N*-dibenzyl- daunomycin	S268242	48	0.238	0.449	inhibitor topoisomerase II

**3.16**	0.514	Batracylin	S320846	43	0.130	0.274	inhibitor of DNA topoisomerases I and II induces histone gamma-H2AX
	0.444	Cyclopentenyl cytosine	S5375575	51	0.122	0.903	CTP synthetase inhibitor (conversion UTP to CTP)

**3.18**	0.460	Hycanthone	S142982	53	0.272	0.220	inducer of nuclear immunoreactivity to antinucleoside antibodies in HeLa cells

a Only correlations with PCC≥ 0.4 were selected, as significant;

b Putative mechanisms of action were identified with the use of literature sources.
